# Design, Synthesis and Evaluation of Novel Phorbazole C Derivatives as MNK Inhibitors through Virtual High-Throughput Screening

**DOI:** 10.3390/md20070429

**Published:** 2022-06-29

**Authors:** Xin Jin, Maowei Li, Tingting Qiu, Rilei Yu, Tao Jiang

**Affiliations:** 1 School of Medicine and Pharmacy, Ocean University of China, Qingdao 266003, China; jinxin1631@ouc.edu.cn (X.J.); 21190831024@stu.ouc.edu.cn (M.L.); qiutingting@stu.ouc.edu.cn (T.Q.); ryu@ouc.edu.cn (R.Y.); 2 Laboratory for Marine Drugs and Bioproducts, Qingdao National Laboratory for Marine, Science and Technology, Qingdao 266237, China

**Keywords:** MNK, eIF4E, phorbazole C, high-throughput screening, structure-activity relationship

## Abstract

MNKs (mitogen-activated protein kinase-interacting protein kinases) phosphorylate eIF4E at Ser209 to control the translation of certain mRNAs and regulate the process of cell proliferation, cell migration and invasion, etc. Development of MNK inhibitors would be an effective treatment for related diseases. We used the MarineChem3D database to identify hit compounds targeting the protein MNK1 and MNK2 through high-throughput screening. Compounds from the phorbazole family showed good interactions with MNK1, and phorbazole C was selected as our hit compound. By analyzing the binding mode, we designed and synthesized 29 derivatives and evaluated their activity against MNKs, of which, six compounds showed good inhibition to MNKs. We also confirmed three interactions between this kind of compound and MNK1, which are vital for the activity. In conclusion, we report series of novel MNK inhibitors inspired from marine natural products and their relative structure–activity relationship. This will provide important information for further developing MNK inhibitors based on this kind of structure.

## 1. Introduction

The MNKs (mitogen-activated protein kinase-interacting protein kinases) can be activated by Erk and p38 to regulate cellular functions [1,2]. MNKs include MNK1 and MNK2, which phosphorylate eIF4E (eukaryotic initiation factor 4E), PSF (polypyrimidine-tract-binding protein-associated splicing factor), cPLA2 (cytoplasmic phospholipase A2), hSPRY2 (sprouty2), and hnRNPA1 (heterogeneous nuclear RNA-binding protein A1) in vitro, but only eIF4E can be phosphorylated at Ser209 in vivo [3,4,5]. eIF4E can bind to the 5′-cap of mRNAs and regulate the translation of certain mRNAs [6,7,8]. The phosphorylation of eIF4E by MNKs is involved in regulating some mRNA translation and related synthesis of proteins, such as MMP-9, SNAIL, and c-MYC [3,8,9]. Overexpression of eIF4E, p-eIF4E and MNKs has been found in various cancers [10,11,12,13], promoting cell proliferation, inducing cell migration and invasion, and regulating tumor microenvironment (TME) by increasing the secretion of proinflammatory cytokines [14,15]. Additionally, MNKs could prevent weight gain and enhance insulin sensitivity and energy expenditure in a high=fat diet [16,17,18]. Moreover, inhibition of MNKs impaired the Toll-like receptor (TLR) signaling pathways [15], cellular Type I and Type II interferon signaling [19,20], and decreased the production of IL-6, IL-10, MCP-1, etc. [21]. Additionally, some researchers have found that MNK is involved the process of drug resistance in cancer. Tamoxifen-resistant estrogen receptor (ER+) breast cancer has a strong positive relationship with increased MNK phosphorylation of eIF4E [22] and rapamycin-resistant KRAS^G12D^ colorectal cancer activated the MNK/eIF4E pathways with overexpression of c-MYC [23].

**Figure 1:**
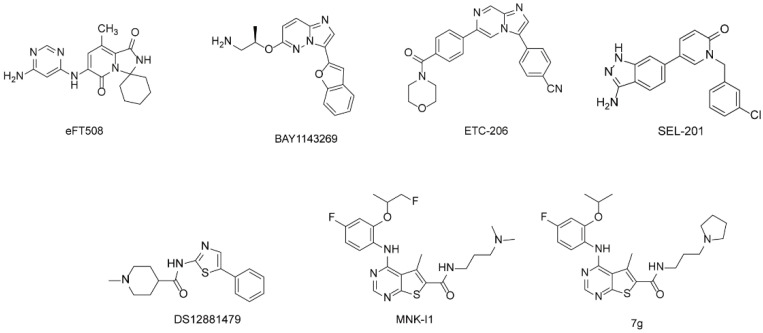
The structure of typical MNK inhibitors that have been reported.

Given that MNKs knock out in mice did not affect the normal phenotype [24], and the phosphorylation of eIF4E is essential for tumor progression [24,25], development of MNK inhibitors would be an effective treatment for related diseases. Although the study of MNK inhibitors has lasted for more than 10 years, none have been approved for market. Inhibitors of CGP57380 [26], staurosporine [27] and cercosporamide as MNKs agents have been found to target other kinases potently [3]. Other MNK inhibitors including MNK-I1 [6], MNK-7g [28], SEL-201 [29], ETC-206 [30], DS12881479 [31], eFT508 [32] and BAY1143269 [33] (Figure 1) have been found that inhibit the activity of MNK1 and/or MNK2 potently and selectively. Currently, BAY1143269, eFT508 and ETC-206 have entered into clinical trials for different settings [4], while BAY1143269 and ETC-206 have been withdrawn or terminated for some reasons (see clinicaltrials.gov accessed on 12 July 2020). We still need more MNK inhibitors with novel structures to interfere with MNK’s pharmacologic action and push the application of MNK inhibitors in more diseases.

Marine natural products possess rich and novel chemical structures owing to the unique ecosystem of the ocean. There are more than 30,000 marine natural small compounds that have been found, while the study of these compounds is limited. Our group participated in the construction of a 3D structure database of these marine natural products (MarineChem3D, http://mc3d.qnlm.ac/, accessed on 10 March 2020) in order to find more active compounds through virtual high-throughput screening. We used this database and docked with the protein complexes of MNK1 (PDB: 5wvd) and MNK2 (PDB: 2hw7). As a result, five hits were chosen by analyzing their interaction mode with MNK1 or MNK2. Phorbazole series compounds, which were found from Aldisa andersoni [34], showed better interaction with the MNK1 protein. We designed and synthesized series derivatives of phorbazole C and evaluated their activity against MNKs. Finally, we discussed their structure–activity relationship (SAR) and found a novel MNK inhibitor. This can be a starting compound for further exploration.

## 2. Results

### 2.1. The Discovery of Hit Compounds 

**Figure 2:**
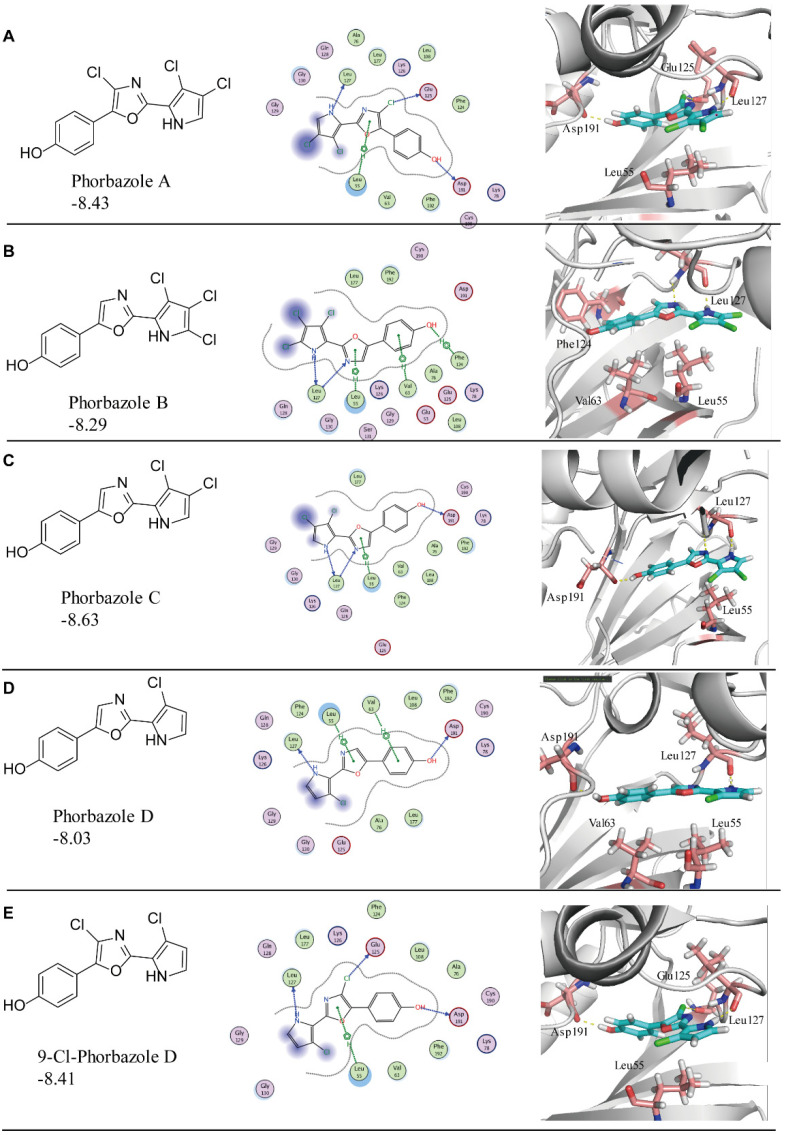
Five compounds are selected through high-throughput screening from MarineChem3D database. (**A**–**E**) show the interaction map between the compound and MNK1 protein (PDB:5wvd), respectively. Compounds are shown in stick representation: carbon in green, nitrogen in blue, oxygen in red, sulfur in yellow, chlorine in dark green. Hydrogen bonds are shown with yellow dashed lines.

Through high-throughput screening of MarineChem3D targeting to MNK1 and MNK2 proteins, we obtained five hit compounds including phorbazole A, phorbazole B, phorbazole C, phorbazole D, and 9-chloro-phorbazole D, which showed good binding to the MNK1 protein complex (PDB: 5wvd) (Figure 2). These compounds belong to phorbazole series, consisting of three parts in the structure, a phenol motif, an oxazole motif and a pyrrole motif. The interaction maps showed that the hydroxyl of benzene formed a hydrogen bond with residue Asp191, and two hydrogen bonds formed between the N atom of oxazole and the NH of pyrrole and residue Leu127 (Figure 2A–E). The 9-chlorine in phorbazole A and 9-chloro-phorbazole D formed a hydrogen bond with residue Glu125 (Figure 2A,D) and the oxazole formed π-H interaction with Leu55. However, other chlorines in pyrrole exposed the outer of the binding pocket (Figure 2B,C), and considering halogen are the characters of marine natural products, we set phorbazole C as the starting point to design our derivatives.

### 2.2. Design and Synthesis of Derivatives

**Scheme 1:**
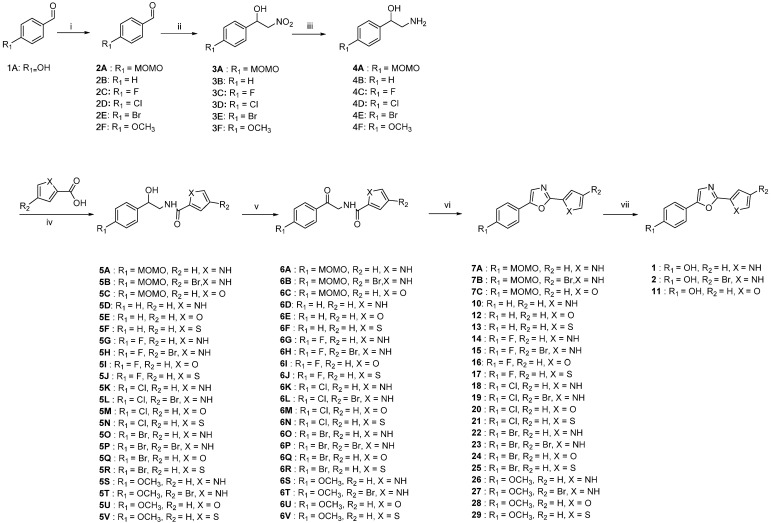
Synthesis of compounds **1**, **2**, **10**–**29**. Reagent and conditions: (i) DIPEA, MOMCl, DCM, r.t.; (ii) MeNO_2_, NEt_3_, DMSO, r.t.; (iii) H_2_, Pd/C, CH_3_OH, r.t.; (iv) Pyrrole-2-carboxylic acid, EDCI, HOBt, DCM, r.t.; (v) DMP, DCM, r.t.; (vi) PPh_3_, C_2_Cl_6_, Et_3_N, CH_2_Cl_2_, r.t.; (vii) HCl, CH_3_OH, 50 °C.

**Scheme 2:**
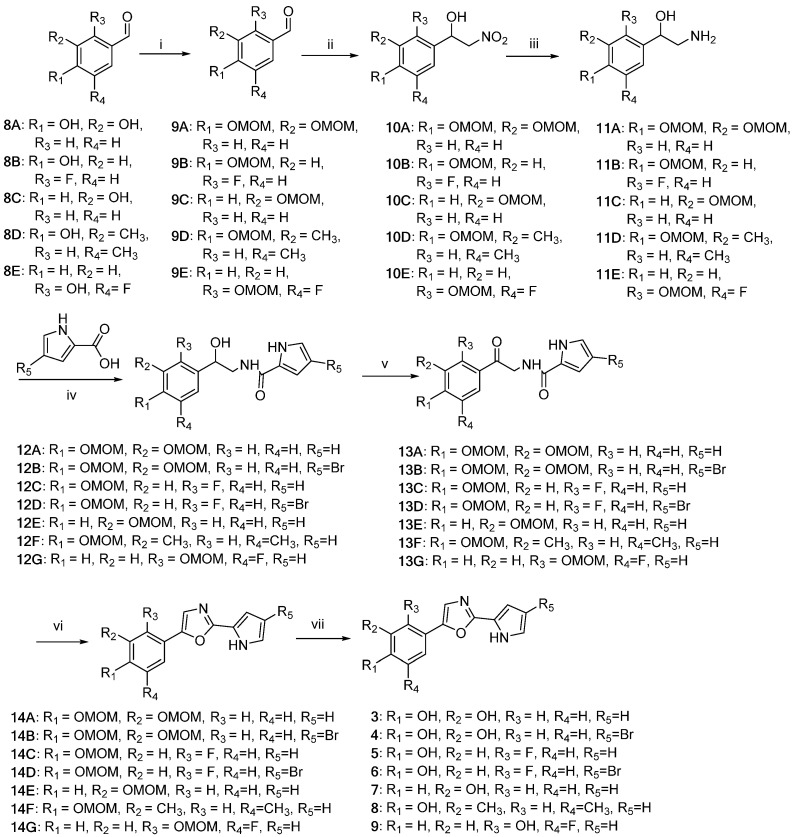
Synthesis of compounds **3**–**9**. Reagent and conditions: (i) DIPEA, MOMCl, DCM, r.t.; (ii) MeNO_2_, NEt_3_, DMSO, r.t.; (iii) H_2_, Pd/C, CH_3_OH, r.t.; (iv) Pyrrole-2-carboxylic acid or 4-bromo-1H-pyrrole-2-carboxylic acid, EDCI, HOBt, DCM, r.t.; (v) DMP, DCM, r.t.; (vi) PPh_3_, C_2_Cl_6_, Et_3_N, CH_2_Cl_2_, r.t.; (vii) HCl, CH_3_OH, 50 °C.

To confirm the role the hydroxyl group and pyrrole played in the inhibition to MNKs activity, we designed three series of compounds: (i) change the position of hydroxyl in the benzene with or without other substituents, (ii) change the pyrrole group into furan or thiophene, (iii) change the hydroxyl group into other atoms or groups (Scheme 1 and Scheme 2).

The total synthetic route of phorbazole C has been reported [35]. According to that, we synthesized compounds **1**–**29**. The hydroxyl groups of compounds (**1A**, **8A**–**8E**) were protected by chloromethyl methyl ether (MOMCl) in the presence of N,N-diisopropylethylamine (DIPEA) in dichloromethane (DCM) at room temperature. Then, compounds (**2A**–**2F**, **9A**–**9E**) were followed by a Henry reaction with nitromethane and triethylamine (Et_3_N) in DMSO to yield compounds **3A**–**3F**, **10A**–**10E**, which were catalytically hydrogenated to the aminoethanol (**4A**–**4F**, **11A**–**11E**) in the presence of Pd/C and hydrogen. The amide compounds (**5A**–**5V**, **12A**–**12G**) were achieved through the reaction of aminoethanol compounds and related acids in the presence of EDCI and HOBt. The acylaminoalcohol compounds were oxidized to the ketone (**6A**–**6V**, **13A**–**13G**) by Dess-Martin periodinane (DMP). Cyclodehydration of the acylaminoketone compounds to the oxazole (**7A**–**7C**, **14A**–**14G**, **10**, **12**–**29**) was realized in the presence of triphenylphosphine, perchloroethane, and Et_3_N. The oxazole compounds were deprotected by treatment with diluted hydrochloric acid to obtain related compounds (**1**–**9** and **11**).

### 2.3. The Structure–Activity Relationship (SAR) of Derivatives

**Figure 3:**
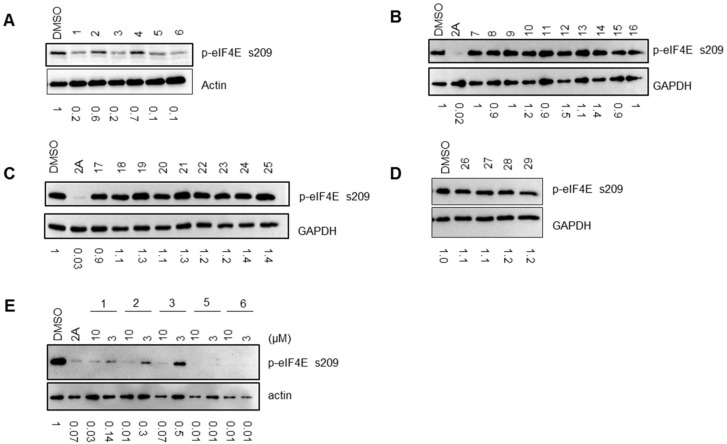
Evaluation of potential MNK inhibitors in 3T3-L1 cells. The cells were treated with the compounds at the indicated concentrations for 2 h, and then cell lysates were analyzed by Western blot for the indicated proteins. (**A**) Mouse 3T3-L1 cells were treated with compound **1**–**6** at 10 µM; (**B**) Mouse 3T3-L1 cells were treated with compound **7**–**16** at 10 µM; (**C**) Mouse 3T3-L1 cells were treated with compound **17**–**25** at 10 µM; (**D**) Mouse 3T3-L1 cells were treated with compound **26**–**29** at 10 µM; (**E**) Mouse 3T3-L1 cells were treated with compound **1**–**3**, **5** and **6** at indicated concentrations. DMSO was used at a final concentration of 0.03–0.05%.

The activity of these compounds against MNKs was tested to assess the level of phosphorylation of eIF4E, which is the best-characterized and only in vivo validated substrate for the MNKs. We used 3T3-L1 cells treated with compounds at 10 µM for 2 h and cell lysates were analyzed by SDS-PAGE. The results showed that compounds **1**, **2**, **3**, **4**, **5**, and **6** had strong inhibition of MNKs (Figure 3A), while other compounds did not exert any inhibition of MNKs (Figure 3B–D). We then tested compounds **1**, **2**, **3**, **5** and **6** at a lower concentration of 3 µM, and compounds **5** and **6** were the best inhibitors among these compounds, showing complete inhibition of p-eIF4E at 3 µM (Figure 3E).

When we kept the structure of oxazole and pyrrole, the R_1_ group in benzene played important roles for the activity of MNKs. The -OH group in benzene was better than other groups (-H, -F, -Cl, -Br, -OCH_3_) in the R_1_ position. For example, compound **1** was better than compounds **10**, **14**, **18**, **22** and **26** which did not show any inhibition of MNKs. When R_1_ was hydrogen or R_2_ or R_3_ was -OH, compounds lost the activity against MNKs (compounds **1**-**6** were better than compounds **7** and **8**). When R_1_ was -OH and other groups were R_2_, R_3_ and/or R_4_, this would decrease the inhibition (compounds **8** and **9** did not show inhibitory activity against MNKs). The substituent in R_5_ has little effect on the inhibitory activity, as in compounds **1** and **2**, **5** and **6**. These results indicated that -OH in R_1_ of compounds was vital for MNKs activity. Next, when we kept -OH in R_1_, pyrrole was replaced by furan and the compound lost the inhibitory activity (compounds **1** and **11**), which means NH in pyrrole was necessary for MNKs inhibitory activity.

**Figure 4:**
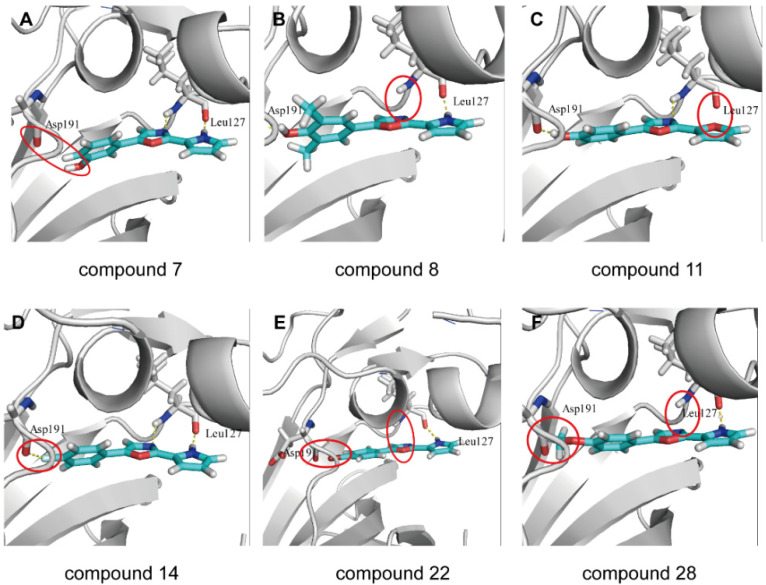
The docking map between indicated compound and MNK1. (**A**–**F**) the interaction maps between the MNK1 and indicated compound. Compounds are shown in stick representation: carbon in green, nitrogen in blue, oxygen in red, sulfur in yellow, bromine in dark red and fluorine in light green. Hydrogen bonds are shown with yellow dashed lines.

We then used the docking software (MOE) to study why these groups are important for the activity against MNKs (Figure 4). The docking map of Phorbazole C showed the hydrogen of -OH formed a hydrogen bond with the oxygen atom of Asp191. When the -OH group was replaced by halogen, this hydrogen bond could not be formed, owing to a further distance (Figure 4A,E). The -OH at other positions of benzene was far away from Asp191, resulting in this hydrogen bond interaction being missed (Figure 4A). When we introduced bulk groups in the benzene, the interaction between oxazole and Leu127 disappeared and the inhibition of MNKs was lost (Figure 4B). When the pyrrole group was replaced by furan, the interactions with Asp191 and Leu127 were kept. However, a hydrogen bond with Leu127 was lost (Figure 4F). All these results indicated that these three hydrogen bonds play vital roles in interacting with MNK1 to show inhibitory activity.

### 2.4. Evaluation the Activity of the Compounds

**Figure 5:**
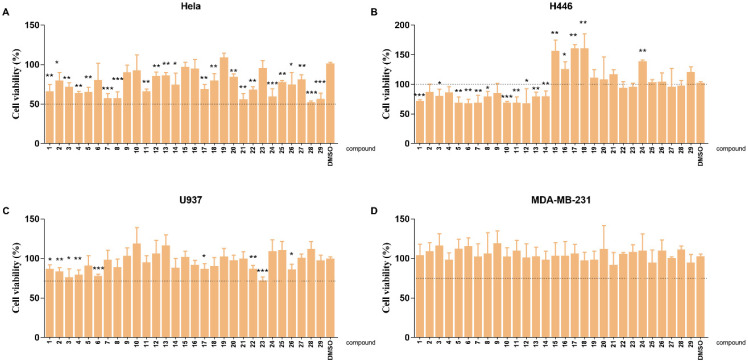
Evaluation of the effects on cell viability of all the compounds at 10 µM. (**A**) The effects on Hela cells. (**B**) The effects on H446 cells. (**C**) The effects on U937 cells. (**D**) The effects on MDA-MB-231 cells. Error bars are SEM. *, *p* < 0.05; **, *p* < 0.005, ***, *p* < 0.0005.

**Figure 6:**
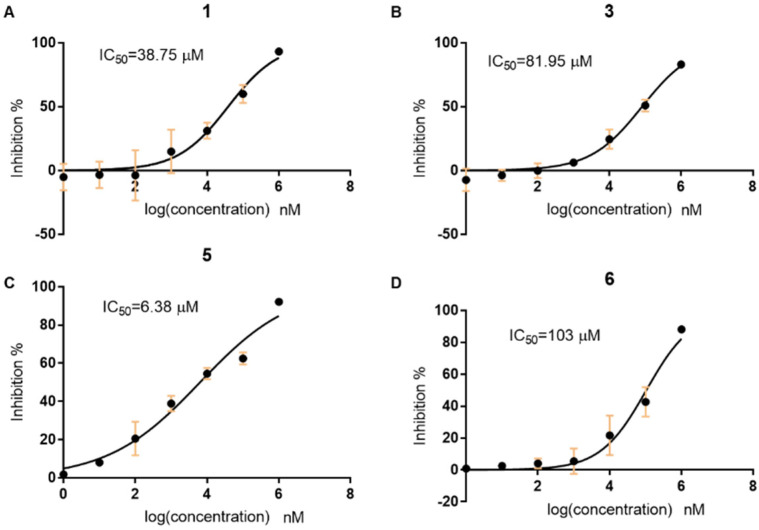
Evaluation the IC_50_ values of indicated compounds in H446 cells. (**A**–**D**) show the concentration–inhibition curve of compounds **1**, **3**, **5**, **6,** respectively.

To evaluate the effects of compounds on cell proliferation, we conducted the MTT assay in H446, Hela, MDA-MB-231 and U937 cell lines. All the compounds at 10 µM showed weak inhibition of the cell viability, including six MNK inhibitors (compounds **1**–**6**) (Figure 5). Among these four cell lines, compounds **1**–**6** showed about 50% inhibition in H446 cells. We then selected four compounds (**1**, **3**, **5** and **6**) to test their IC_50_ values using MTT assays. Compound **5** had a higher cytotoxic activity, with an IC_50_ of 6.38 µM (Figure 6).

**Figure 7:**
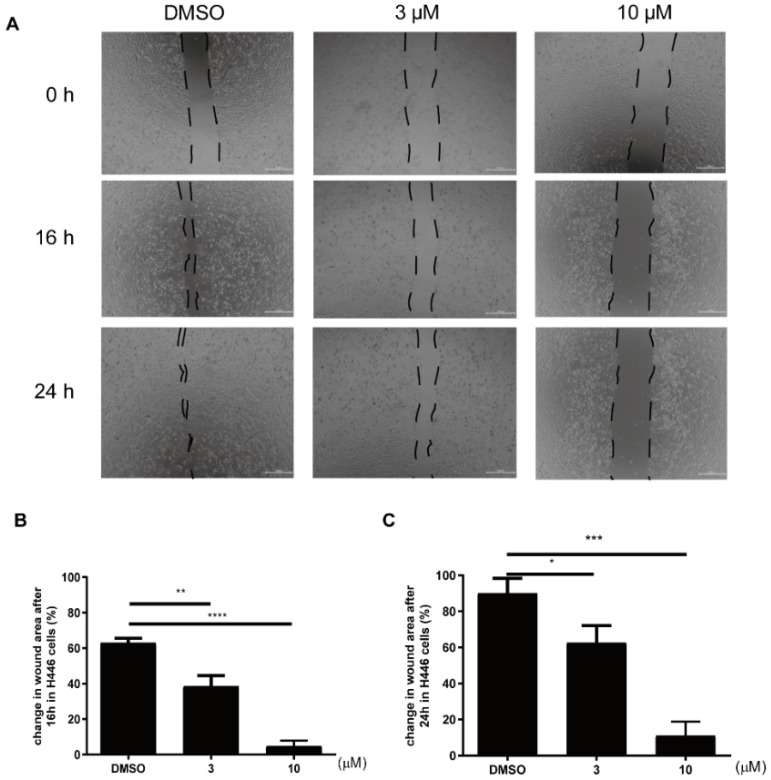
Compound **5** inhibits cell migration of H446 cells in ‘wound-healing’ assays. (**A**) Scratch wound-healing assays were performed for H446 cells treated with 5 (10 μM and 3 μM) or DMSO for the indicated times. Pictures were taken at the indicated time points. (**B**) Percentage change in wound area after 16 h was analyzed by Image J. (**C**) Percentage change in wound area after 24 was analyzed by Image J. *, *p* < 0.05; **, *p* < 0.005, ***, *p* < 0.0005, ****, *p* < 0.0001.

The inhibition of MNKs and the phosphorylation of eIF4E have been found to impair the process of EMT (epithelial-to-mesenchymal transition) and metastasis in cancer cells. Therefore, we assessed the influence of compound **5** on H446 cells. We used ‘scratch wound-healing assays’ to analyze the effect of **5** on the migratory potential of H446 cells. In line with the previous report, we observed significantly reduced migratory potential in cells with **5** compared with DMSO at 16 h after treatment (Figure 7A,B), and the effect was even more pronounced at 24 h (Figure 7A,C).

## 3. Discussion

MNKs regulate cell functions by involving the process of mRNA translation and protein synthesis through the phosphorylation of eIF4E [36]. Inhibitory activity of MNKs and then the phosphorylation of eIF4E could be an effective and safe target for the treatment of cancers. MNK inhibitors have been developed for several years; however, none have been approved. Most inhibitors showed ‘off target’ effects, inhibiting other kinases potently [3]. Therefore, selective inhibitors with novel structure are still needed.

Marine natural products possess novel chemical structures with good bioactivity, while most compounds have not found value in medicine. The development and utilization of these compounds is necessary. MNK1/2 kinases belong to the family of Ca^2+^/calmodulin-dependent kinases (CaMKs) and share 80% homology with other kinases, except for a unique DFD-motif and three insertions [37]. These characteristics are the key points for the design of inhibitors. In this article, we used virtual high-throughput screening technology targeting the MNK1 and MNK2 protein complex based on the MarineChem3D database that comprises more than 30,000 marine natural products. Five compounds (phorbazole A, phorbazole B, phorbazole C, phorbazole D, and 9-chloro-phorbazole D) were selected for their good binding interactions with MNK1. We selected phorbazole C as a hit compound and designed a series of derivatives by analyzing the interaction mode between phorbazole C and MNK1. Six compounds showed good activity against MNKs and inhibited the phosphorylation of eIF4E in 3T3-L1 cells. Using the docking software MOE and pymol, we analyzed and concluded how the groups in this structure affect the MNK inhibitory activity. Firstly, the hydroxyl group at the para position of benzene formed a hydrogen bond with Asp191. Other groups or atoms (such as -F, -Br, -Cl and -OCH_3_) in this position could decrease or lose their inhibition of MNKs owing to this important hydrogen bond not being formed. We also found that multi-substituted phenol would decrease the inhibition. Secondly, the oxazole and pyrrole also showed two hydrogen bonds’ formation with Lue127 of the hinge region in the MNK1 pocket. Substituents in pyrrole also decrease the inhibitory activity of MNKs, and these two interactions seem be vital for this activity. The results showed that these hydrogen bonds keep the compound stable in the pocket, which provides an idea for further designing derivatives and selective MNK inhibitors. 

Furthermore, we also found if we use the amino group (-NH_2_) to replace the hydroxyl group at the para position of benzene, it would retain all these interactions, and may show MNK-inhibitory activity. Unfortunately, we did not obtain this compound in our synthetic route. In future, we still need to confirm this result by an appropriate route to obtain better MNK inhibitors with novel structure and good selectivity.

Several sources have reported that overexpression of MNKs and p-eIF4E were associated with the progression and development of cancer [38,39]. The loss of MNK1/2 genes did not affect cell proliferation in MEFs and KIT-mutant melanoma cells [16,25], but inhibiting the activity of MNK2 in non-small-cell lung cancer impaired the proliferation and migration [40]. In our assay, compounds **1**–**6** showed weak effects on cell proliferation in Hela, U937 and MDA-MB-231 cell lines, but good inhibition rate in H446 cells. Compound **5** also inhibited the migration of H446 cells significantly, which was in accordance with a previous report. Novel and potent MNK inhibitors could be designed and synthesized based on this structure.

## 4. Materials and Methods

### 4.1. Reagents and Tumor Cell Lines

For in vitro experiments, all compounds were dissolved in dimethyl sulfoxide (DMSO). All the cell lines were obtained from Procell (Wuhan, China). 3T3-L1 and MDA-MB-231 were cultured in Dulbecco’s modified Eagle media (DMEM) with high glucose (4500 mg/L) with pyruvate supplemented with 10% fetal bovine serum (FBS) and 1% penicillin–streptomycin. H446 and U937 cells were culture in Roswell Park Memorial Institute-1640 (RPMI-1640) media (2 g/L glucose) containing 10% FBS and 1% penicillin/streptomycin.

### 4.2. SDS-PAGE and Western Blot

Cell monolayers were harvested in RIPA lysis buffer with 1 mM PMSF from a commercial company (Beyotime, Beijing China). After lysis, insoluble material was removed by centrifugation at >12,000× *g* for 10 min at 4 °C. Protein content was determined by the BAC assay. Cell lysates were heated at 95 °C for 3 min in sample buffer (250 mM Tris–HCl, 10% (*v/v*) sodium dodecyl sulfate (SDS), 20% (*v/v*) glycerol, 0.12% (*w/v*) bromophenol blue) and subjected to polyacrylamide gel electrophoresis (PAGE) and electrophoretic transfer to nitrocellulose membranes. Membranes were then blocked in phosphate-buffered saline (PBS)-0.05% Tween20 containing 5% (*w/v*) skim milk powder for 30 min at room temperature. Membranes were probed with the indicated primary antibody overnight at 4 °C. After incubation with HRP-tagged secondary antibody, blots were visualized using Tanon-5200. Primary antibodies and secondary antibodies were from Abcam.

### 4.3. Scratch Wound-Healing Assay

Cells were seeded on 24-well plates. When the cells had reached 90–100% confluence, wounds to cell layers were induced by scratching with a 10 μL pipette tip. The medium was changed to remove any floating cells and the cells were then treated with compound **5** (10 μM and 3 μM) or vehicle control (DMSO) in fresh medium for indicated time. Images of wound closure were taken directly by a microscope after scratching as the pre-migration time point and at the indicated time points. Image J software was used to quantify the area of wounds (open area).

### 4.4. MTT Assay

Cell viability was tested by 3-(4,5-dimethylthiazol-2-yl)-2,5-diphenyltetrazolium bromide (MTT) assay. Cells were seeded on 96-well cell culture plates with or without inhibitor at indicated concentrations in 200 µL of complete growth medium. After the indicated time, 20 µL of 5 mg/mL MTT solution was added to each well. Four hours later, 150 mL DMSO was added to each well and the cells were incubated for a further 15–30 min. Absorbance of the solubilized MTT was then measured using the SpectraMax i3 at 490 nm.

### 4.5. Molecular Docking

Molecular docking was performed using MOE (Molecular Operating Environment) with AMBER10: EHT forcefield. The crystal structure of MNK1 was selected and downloaded from the Protein DataBank (PDB, http://www.rcsb.org, accessed on 12 July 2020), and was used for docking. The induced-fit docking approach was applied with consideration of the side-chain flexibility of residues at the binding site. The ligand binding site was defined using the bound ligands in the crystal structures. The best scored conformation with minimum binding energy from the 30 docking conformations of the ligands was selected for analysis

### 4.6. General Methods for Synthetic Chemistry 

All starting materials and solvents were obtained from commercial sources and used without further purification. All actions were carried out with continuous magnetic stirring in common glassware and heating of reactions was performed with an IKA® heating block. Thin-layer chromatography (TLC) was performed on precoated silica-gel 60 F254 plates (E. Merck, Shanghai, China). Column chromatography was performed on silica gel (200–300 mesh, Qingdao Marine Chemical Company, Qingdao, China). ^1^H NMR and ^13^C NMR spectra were obtained on a Bruker 400 NMR spectrometer (^1^H at 400 MHz and ^13^C at 100 MHz) with tetramethylsilane (Me_4_Si) as the internal standard. Chemical shifts are reported as δ values. Mass spectra were recorded on a Q–TOF Global mass spectrometer.

All the compounds were synthesized according to previous report [35]. The data of ^1^H and ^13^C NMR and the spectra were shown in Supplementary Materials.

#### 4.6.1. General Synthesis Method for Compounds **2A** and **9A**–**9E**

Compounds **1A** or **8A**–**8E** (1 eq) and DIPEA (3 eq) were mixed in DCM, and MOMCl (3 eq) was dropwise added to the solution at 0 °C. The mixture was stirred for 8–10 h at room temperature. After the reaction was judged complete by TLC (PE:EA = 20:1), the mixture was quenched by saturated NaHCO_3_ and extracted with DCM three times. The combined organic layers were washed with water and brine, dried over anhydrous MgSO_4_, filtered, and concentrated in vacuo. The crude product was purified by chromatography (PE:EA = 20:1) to yield compounds **2A** and **9A**–**9E**.

#### 4.6.2. General Synthesis Method for **3A**–**3F** and **10A**–**10E**

A mixture of **2A**–**2F** or **9A**–**9E** (1 eq), nitromethane (3 eq) and Et_3_N (1 eq) in DMSO was stirred at room temperature for 12 h. The mixture was poured into a saturated aqueous NH_4_Cl solution and adjusted to pH 7 by adding hydrochloric acid. The mixture was extracted with DCM three times. The combined organic layers were washed with water and brine, dried over anhydrous MgSO_4_, filtered, and concentrated in vacuo. The crude product was purified by chromatography (PE:EA = 10:1) to yield compounds **3A**–**3F** and **10A**–**10E**.

#### 4.6.3. General Synthesis Method for **4A**–**4F** and **11A**–**11E**

A mixture of **3A**–**3F** or **10A**–**10E** and moderate 10% Pd/C in methanol under a hydrogen balloon was stirred at room temperature overnight. The reaction mixture was filtered and washed with methanol. The combined filtrate was concentrated in vacuo and the crude product was used for the next step without further purification.

#### 4.6.4. General Synthesis Method for **5A**–**5V** and **12A**–**12G**

The related carboxylic compound (1 eq) was mixed with EDCI (2 eq) and HOBt (2 eq) in DCM at room temperature for 8 h. Then, **4A**–**4F** or **11A**–**11E** (1 eq) was added to the mixture for another 3 h. The mixture was washed with water and extracted with DCM three times. The combined organic layers were washed with water and brine, dried over anhydrous MgSO_4_, filtered, and concentrated in vacuo. The crude product was purified by chromatography (PE:EA = 1:1) to yield compounds **5A**–**5V** and **12A**–**12G**.

#### 4.6.5. General Synthesis Method for **6A**–**6V** and **13A**–**13G**

To a solution of **5A**–**5V** or **12A**–**12G** (1 eq) in dry DCM, Dess–Martin periodinane (1.11 eq) was added. The mixture was stirred at room temperature until the reactant disappeared and quenched by adding solution of Na_2_S_2_O_3_ and saturated aqueous NaHCO_3_. The organic layer was separated and the aqueous phase was extracted with DCM three times. The combined organic layers were washed with water and brine, dried over anhydrous MgSO_4_, filtered, and concentrated in vacuo. The crude product was purified by chromatography (PE:EA = 1:1) to yield compounds **6A**–**6V** and **13A**–**13G**.

#### 4.6.6. General Synthesis Method for **7A**–**7C**, **10**, **12**–**29** and 1**4A**–**14G**

The mixture of **6A**–**6V** or **14A**–**14G** (1 eq), thiphenylphosphine (3 eq), hexachloroethane (6 eq) and triethylamine (6 eq) in DCM was stirred for 12 h at room temperature. The mixture was washed by water and extracted by DCM three times. The combined organic layers were washed with water and brine, dried over anhydrous MgSO_4_, filtered, and concentrated in vacuo. The crude product was purified by chromatography (PE:EA=2:1) to yield compounds **7A**–**7C, 10, 12**–**29** and **14A**–**14G**.

#### 4.6.7. General Synthesis Method for **1**–**9** and **11**

Compounds **7A**–**7C, 10, 12**–**29** or **14A**–**14G** were dissolved in methanol and HCl (1 mL) was added dropwise. The mixture was stirred for 1 h at 50 °C. After the reaction was judged complete by TLC (PE:EA = 1:1), the mixture was concentrated in vacuo and extracted with DCM three times. The combined organic layers were washed with water and brine, dried over anhydrous MgSO_4_, filtered, and concentrated in vacuo. The crude product was purified by chromatography (PE:EA = 1:1) to yield compounds **1**–**9** and **11**.

### 4.7. Statistics

All in vitro experiments were performed in triplicate, and quantitative data are shown as the average of all biological replicates. All statistical analyses were performed using GraphPad Prism 7 (Version 7.0, GraphPad Software, San Diego, CA, USA). For simple comparisons, an unpaired two-tailed Student’s *t* test was used.

## 5. Conclusions

In conclusion, we obtained six MNK inhibitors by virtual high-throughput screening and evaluated their effect on phosphorylation of eIF4E. Among these inhibitors, compound **5** with the skeleton of oxazole and pyrrole derived from phorbazole C showed the best inhibitory activity against MNKs. Combining the interaction mode between compounds and MNK1, we showed that three hydrogen bonds (one hydrogen bond with Asp191, two hydrogen bonds with Leu127) were important for the inhibition of MNKs. In MTT assays, all compounds showed weak inhibition to cell proliferation in these cancer cell lines, and only compound **5** exerted better cytotoxic activity with an IC_50_ of 6.38 μM in H446 cells. Additionally, our inhibitor (compound **5**) inhibited the migration of H446 cells significantly by impairing the MNK/eIF4E axis. Further work should be carried out to exploit its pharmacology activity and to design more novel inhibitors based on this skeleton.

## Data Availability

Not applicable.

## Supplementary Materials

The following supporting information can be downloaded at: https://www.mdpi.com/article/10.3390/md20070429/s1, The data of ^1^H and ^13^C NMR and the spectra of all the compounds (**1**–**29**) are shown in Supplementary Materials.
