# Supplementary material for: Design, Synthesis and Evaluation of Novel Phorbazole C Derivatives as MNK Inhibitors through Virtual High-Throughput Screening
Source: Mar Drugs. 2022 Jun 29;20(7):429. doi: 10.3390/md20070429 (PMC9319845; doi:10.3390/md20070429)
Supplement: Supplementary file 1 [file marinedrugs-20-00429-s001.zip › marinedrugs-1781619-supplementary.pdf]

Supplementary Materials

4-(2-(1H-pyrrol-2-yl)oxazol-5-yl)phenol (**1**)

Compound **7A** (158 mg, 0.59 mmol) and HCl (215 μL, 5.9 mmol) were mixed in methanol. Following the general method, compound **1** was got as yellow solid (134 mg) in a 35% yield. Melting point: 218-219 °C. ^{1}H NMR (400 MHz, DMSO-d$_6$) δ 11.81 (s, 1H), 9.90 (s, 1H), 7.62 – 7.55 (m, 2H), 7.44 (s, 1H), 6.92 – 6.84 (m, 2H), 6.62 (t, *J* = 2.9 Hz, 1H), 5.98 (t, *J* = 2.8 Hz, 1H), 3.99 (s, 1H); ^{13}C NMR (100 MHz, DMSO-d$_6$) δ 157.27, 154.63, 149.72, 125.82, 122.99, 117.12, 111.74, 96.58, 94.28, 56.17; HRMS calcd for $(C_{13}H_{10}N_2O_2 + H)^+$ 227.0742, found 227.0740.

4-(2-(4-bromo-1*H*-pyrrol-2-yl)oxazol-5-yl)phenol (**2**)

Compound **7B** (100 mg, 0.29 mmol) and HCl (106 μL, 2.9 mmol) were mixed in methanol. Following the general method, compound **2** was got as yellow solid (48 mg) in a 49% yield. Melting point: 238 °C. ^{1}H NMR (400 MHz, DMSO-*d$_6$*) *δ* 12.25 (s, 1H), 9.81 (s, 1H), 7.67 – 7.43 (m, 3H), 7.13 (s, 1H), 6.97 – 6.69 (m, 3H); ^{13}C NMR (100 MHz, DMSO-*d$_6$*) *δ* 158.24, 154.23, 150.30, 126.03, 122.07, 121.48, 121.25, 119.10, 116.31, 111.49, 96.52; HRMS calcd for $(C_{13}H_9BrN_2O_2 + H)^+$ 304.9847, found 304.9843.

4-(2-(1*H*-pyrrol-2-yl)oxazol-5-yl)benzene-1,2-diol (**3**)

Compound **14A** (110 mg, 0.34 mmol) and HCl (124 μL, 3.4 mmol) were mixed in methanol. Following the general method, compound **3** was got as yellow solid (33 mg) in a 40% yield. Melting point: 233 °C. ^{1}H NMR (400 MHz, DMSO-*d$_6$*) *δ* 11.86 (s, 1H), 9.25 (d, *J* = 70.3 Hz, 2H), 7.40 (s, 1H), 7.14 (d, *J* = 2.1 Hz, 1H), 7.07 (dd, *J* = 8.2, 2.1 Hz, 1H), 6.97 (q, *J* = 2.2 Hz, 1H), 6.83 (d, *J* = 8.2 Hz, 1H), 6.71 (p, *J* = 1.8 Hz, 1H), 6.21 (q, *J* = 2.7 Hz, 1H); ^{13}C NMR (100 MHz, DMSO-*d$_6$*) *δ* 154.13, 150.50, 146.40, 122.16, 121.21, 119.37, 116.60, 116.23, 112.08, 111.51, 96.53; HRMS calcd for $(C_{13}H_{10}N_2O_3 + H)^+$ 242.0691, found 242.0683.

4-(2-(4-bromo-1*H*-pyrrol-2-yl)oxazol-5-yl)benzene-1,2-diol (**4**)

Compound **14B** (108 mg, 0.24 mmol) and HCl (88 μL, 2.4 mmol) were mixed in methanol. Following the general method, compound **4** was got as yellow solid (37 mg) in a 48% yield. Melting point: >240 °C. ^{1}H NMR (400 MHz, DMSO-*d$_6$*) *δ* 12.28 (t, *J* = 2.8 Hz, 1H), 8.88 (s, 2H), 7.45 (s, 1H), 7.16 (d, *J* = 2.1 Hz, 1H), 7.13 (dd, *J* = 2.9, 1.7 Hz, 1H), 7.08 (dd, *J* = 8.2, 2.1 Hz, 1H), 6.85 (d, *J* = 8.2 Hz, 1H), 6.78 (dd, *J* = 2.6, 1.6 Hz, 1H); ^{13}C NMR (100 MHz, DMSO-*d$_6$*) *δ* 154.13, 150.50, 146.40, 122.12, 121.27, 119.35, 116.68, 116.22, 112.14, 111.45, 96.50; HRMS calcd for $(C_{13}H_9BrN_2O_3 + H)^+$ 320.9797, found 320.9790.

4-(2-(1*H*-pyrrol-2-yl)oxazol-5-yl)-3-fluorophenol (**5**)

Compound **14C** (90 mg, 0.31 mmol) and HCl (114 μL, 3.1 mmol) were mixed in methanol. Following the general method, compound **5** was got as yellow solid (60 mg) in a 79% yield. Melting point: >240 ^{o}C. ^{1}H NMR (400 MHz, DMSO-*d*$_6$) *δ* 11.93 (s, 1H), 10.30 (s, 1H), 7.71 (t, *J* = 8.7 Hz, 1H), 7.31 (d, *J* = 3.7 Hz, 1H), 7.01 (td, *J* = 2.6, 1.4 Hz, 1H), 6.85 – 6.68 (m, 3H), 6.22 (q, *J* = 2.6 Hz, 1H); ^{13}C NMR (100 MHz, DMSO-*d*$_6$) *δ* 160.54, 159.38, 158.07, 155.58, 144.15, 127.20, 124.57, 122.63, 119.97, 112.80, 110.30, 107.43, 103.74；HRMS calcd for $(C_{13}H_9FN_2O_2 + H)^+$ 245.0648, found 245.0642.

4-(2-(4-bromo-1*H*-pyrrol-2-yl)oxazol-5-yl)-3-fluorophenol (**6**)

Compound **14D** (100 mg, 0.27 mmol) and HCl (100 μL, 2.7 mmol) were mixed in methanol. Following the general method, compound **6** was got as grey solid (65 mg) in a 69% yield. Melting point: >240 ^{o}C. ^{1}H NMR (400 MHz, DMSO-*d*$_6$) *δ* 12.33 (t, *J* = 2.8 Hz, 1H), 10.34 (s, 1H), 7.73 (t, *J* = 8.7 Hz, 1H), 7.35 (d, *J* = 3.7 Hz, 1H), 7.17 (dd, *J* = 2.9, 1.7 Hz, 1H), 6.85 (dd, *J* = 2.6, 1.7 Hz, 1H), 6.81 – 6.67 (m, 2H); ^{13}C NMR (100 MHz, DMSO-*d*$_6$) *δ* 160.63, 159.61, 158.16, 154.23, 144.73, 127.39, 124.60, 122.40, 120.96, 112.37, 107.19, 103.73, 96.63, 60.23, 21.24, 14.56; HRMS calcd for $(C_{13}H_8BrFN_2O_2 + H)^+$ 322.9753, found 322.9748.

3-(2-(1*H*-pyrrol-2-yl)oxazol-5-yl)phenol (**7**)

Compound **14E** (107 mg, 0.4 mmol) and HCl (146 μL, 4 mmol) were mixed in methanol. Following the general method, compound **7** was got as yellow solid (36 mg) in a 40% yield. Melting point: 222 ^{o}C. ^{1}H NMR (400 MHz, DMSO-*d*$_6$) *δ* 11.94 (s, 1H), 9.68 (s, 1H), 7.64 (s, 1H), 7.32 – 7.11 (m, 3H), 7.01 (q, *J* = 2.2 Hz, 1H), 6.77 (dd, *J* = 7.5, 2.9 Hz, 2H), 6.23 (q, *J* = 2.6 Hz, 1H); ^{13}C NMR (100 MHz, DMSO-*d*$_6$) *δ* 157.33, 149.33, 129.95, 123.84, 122.65, 120.06, 115.37, 111.80, 107.59；HRMS calcd for $(C_{13}H_{10}N_2O_2 + H)^+$ 227.0742, found 227.0733.

4-(2-(1*H*-pyrrol-2-yl)oxazol-5-yl)-2,6-dimethylphenol (**8**)

Compound **14F** (120 mg, 0.4 mmol) and HCl (146 μL, 4 mmol) were mixed in methanol. Following the general method, compound **8** was got as yellow solid (60 mg) in a 55% yield. Melting point: 230-232 ^{o}C. ^{1}H NMR (400 MHz, DMSO-*d*$_6$) *δ* 11.84 (s, 1H), 8.59 (d, *J* = 5.8 Hz, 1H), 7.47 – 7.39 (m, 1H), 7.37 (d, *J* = 6.5 Hz, 2H), 7.05 – 6.92 (m, 1H), 6.81 – 6.71 (m, 1H), 6.21 (dq, *J* = 5.6, 2.6 Hz, 1H), 2.28 – 2.18 (m, 6H); ^{13}C NMR (100 MHz, DMSO-*d*$_6$) *δ* 155.54, 153.99, 149.90, 125.34, 124.43, 122.21, 121.38, 120.32, 119.37, 109.97, 17.1; HRMS calcd for $(C_{15}H_{14}N_2O_2 + H)^+$ 255.1055, found 255.1051.

2-(2-(1H-pyrrol-2-yl)oxazol-5-yl)-4-fluorophenol (**9**)

Compound **14G** (102 mg, 0.38 mmol) and HCl (140 μL, 3.8 mmol) were mixed in methanol. Following the

general method, compound **9** was got as yellow solid (48 mg) in a 52% yield. Melting point: >240 °C. ^{1}H NMR (400 MHz, DMSO) δ 11.93 (s, 1H), 10.30 (s, 1H), 7.71 (t, *J* = 8.7 Hz, 1H), 7.31 (d, *J* = 3.7 Hz, 1H), 7.02 – 6.99 (m, 1H), 6.80 – 6.71 (m, 3H), 6.23 – 6.20 (m, 1H).

5-phenyl-2-(1*H*-pyrrol-2-yl)oxazole (**10**)

Compound **6D** (342 mg, 1.5 mmol), PPh_3 (1.57 g, 6 mmol), C_2Cl_6 (1.24 g, 5.3 mmol) and Et_3N (1.6 mL, 12 mmol) were mixed in DCM. Following the general method, compound **10** was got as yellow solid (110 mg) in a 35% yield. Melting point: >240 °C. ^{1}H NMR (400 MHz, DMSO-*d*6) *δ* 10.69 (s, 1H), 7.53 (s, 2H), 7.51 – 7.47 (m, 2H), 7.46 (s, 1H), 7.16 (s, 1H), 6.96 (s, 1H), 6.53 (s, 2H); ^{13}C NMR (100 MHz, DMSO-*d*6) *δ* 169.00, 161.13, 145.68, 138.52, 133.33, 131.21, 129.13, 128.18, 127.04, 126.17, 66.84; HRMS calcd for ($C_{13}H_{10}N_2O$ + H)$^+$ 211.0793, found 211.0791.

4-(2-(furan-2-yl)oxazol-5-yl)phenol (**11**)

Compound **7C** (80 mg, 0.3 mmol) and HCl (110 μL, 3 mmol) were mixed in methanol. Following the general method, compound **11** was got as yellow solid (40 mg) in a 79% yield. Melting point: 78 °C. ^{1}H NMR (400 MHz, DMSO-*d*6) *δ* 9.90 (s, 1H), 7.62 – 7.55 (m, 2H), 7.44 (s, 1H), 6.92 – 6.84 (m, 2H), 6.62 (t, *J* = 2.9 Hz, 1H), 5.98 (t, J = 2.8 Hz, 1H), 3.99 (s, 1H).

2-(furan-2-yl)-5-phenyloxazole (**12**)

Compound **6E** (153 mg, 0.67 mmol), PPh_3 (0.7 g, 2.67 mmol), C_2Cl_6 (0.5 g, 2.34 mmol) and Et_3N (0.7 mL, 5.36 mmol) were mixed in DCM. Following the general method, compound **12** was got as yellow solid (70 mg) in a 49% yield. Melting point: 67 °C. ^{1}H NMR (400 MHz, DMSO-*d*6) δ 8.02 – 7.96 (m, 1H), 7.83 (dt, *J* = 18.1, 6.8 Hz, 3H), 7.51 (p, *J* = 7.3 Hz, 2H), 7.45 – 7.38 (m, 1H), 7.27 (q, *J* = 4.2 Hz, 1H), 6.77 (d, *J* = 7.9 Hz, 1H); ^{13}C NMR (100 MHz, DMSO-*d*6) δ 153.61, 150.63, 146.08, 142.51, 129.60, 129.15, 127.59, 124.45, 112.71; HRMS calcd for ($C_{13}H_9NO_2$ + H)$^+$ 212.0633, found 212.0628.

5-phenyl-2-(thiophen-2-yl)oxazole (**13**)

Compound **6F** (130 mg, 0.53 mmol), PPh_3 (0.55 g, 2.12 mmol), C_2Cl_6 (0.45 g, 1.9 mmol) and Et_3N (0.6 mL, 4.24 mmol) were mixed in DCM. Following the general method, compound **13** was got as yellow solid (51 mg) in a 43% yield. Melting point: 65 °C. ^{1}H NMR (400 MHz, DMSO-*d*6) δ 7.84 (dd, *J* = 8.8, 4.6 Hz, 5H), 7.52 (t, *J* = 7.6 Hz, 2H), 7.41 (t, *J* = 7.5 Hz, 1H), 7.28 (t, *J* = 4.4 Hz, 1H); ^{13}C NMR (100 MHz, DMSO-*d*6) δ 157.04, 150.71, 130.14, 129.55, 129.02, 128.58, 127.67, 124.46; HRMS calcd for ($C_{13}H_9NOS$ + H)$^+$ 228.0405, found 228.0401.

5-(4-fluorophenyl)-2-(1*H*-pyrrol-2-yl)oxazole (**14**)

Compound **6G** (280 mg, 1.14 mmol), PPh_3 (1.19 g, 4.56 mmol), C_2Cl_6 (0.95 g, 3.9 mmol) and Et_3N (1.26 mL, 9.12 mmol) were mixed in DCM. Following the general method, compound **14** was got as yellow solid (99 mg) in a 38% yield. Melting point: >240 °C. ^{1}H NMR (400 MHz, DMSO-*d*6) *δ* 11.91 (s, 1H), 7.79 (t, *J* = 7.0 Hz, 2H), 7.64 (s, 1H), 7.29 (t, *J* = 8.7 Hz, 2H), 6.98 (s, 1H), 6.76 (s, 1H), 6.19 (s, 1H); ^{13}C NMR (100 MHz, DMSO-*d*6) δ 194.64, 158.58, 146.85, 139.03, 134.01, 129.88, 113.41, 46.29; HRMS calcd for $(C_{13}H_9FN_2O + H)^+$ 229.0699, found 229.0692.

2-(4-bromo-1*H*-pyrrol-2-yl)-5-(4-fluorophenyl)oxazole (**15**)

Compound **6H** (240 mg, 0.74 mmol), PPh_3 (0.77 g, 2.96 mmol), C_2Cl_6 (0.61 g, 2.59 mmol) and Et_3N (0.8 mL, 5.92 mmol) were mixed in DCM. Following the general method, compound **15** was got as yellow solid (120 mg) in a 51% yield. Melting point: >240 °C. ^{1}H NMR (400 MHz, DMSO-*d*6) *δ* 12.32 (s, 1H), 7.92 – 7.80 (m, 2H), 7.72 (s, 1H), 7.35 (t, *J* = 8.8 Hz, 2H), 7.17 (dd, *J* = 3.0, 1.6 Hz, 1H), 6.86 (t, *J* = 2.0 Hz, 1H); ^{13}C NMR (100 MHz, DMSO-*d*6) *δ* 162.34, 155.11, 148.94, 126.49, 124.65, 123.80, 122.45, 120.96, 116.61, 112.03, 96.66; HRMS calcd for $(C_{13}H_8BrFN_2O + H)^+$ 305.9804, found 305.9801.

5-(4-fluorophenyl)-2-(furan-2-yl)oxazole (**16**)

Compound **6I** (192 mg, 0.78 mmol), PPh_3 (0.82 g, 3.12 mmol), C_2Cl_6 (0.65 g, 2.73 mmol) and Et_3N (0.86 mL, 6.24 mmol) were mixed in DCM. Following the general method, compound **16** was got as yellow solid (89 mg) in a 49% yield. Melting point: 83 °C. ^{1}H NMR (400 MHz, DMSO-*d*6) *δ* 7.96 (dd, *J* = 1.8, 0.8 Hz, 1H), 7.87 – 7.74 (m, 3H), 7.38 – 7.29 (m, 2H), 7.24 (dd, *J* = 3.5, 0.8 Hz, 1H), 6.74 (dd, *J* = 3.5, 1.8 Hz, 1H); ^{13}C NMR (100 MHz, DMSO-*d*6) *δ* 162.53, 153.57, 149.82, 146.06, 142.46, 126.74, 124.64,122.77, 116.68, 112.69; HRMS calcd for $(C_{13}H_8FNO_2 + H)^+$ 230.0539, found 230.0532.

5-(4-fluorophenyl)-2-(thiophen-2-yl)oxazole (**17**)

Compound **6J** (122 mg, 0.46 mmol), PPh_3 (0.48 g, 1.84 mmol), C_2Cl_6 (0.38 g, 1.61 mmol) and Et_3N (0.51 mL, 3.68 mmol) were mixed in DCM. Following the general method, compound **17** was got as yellow solid (62 mg) in a 56% yield. Melting point: 79 °C. ^{1}H NMR (400 MHz, DMSO-*d*6) *δ* 7.96 – 7.67 (m, 5H), 7.46 – 7.18 (m, 3H); ^{13}C NMR (100 MHz, DMSO-*d*6) *δ* 163.74, 161.29, 157.02, 149.92, 130.22, 129.45, 128.84, 126.73, 124.34, 116.70; HRMS calcd for $(C_{13}H_8FNOS + H)^+$ 246.0311, found 246.0305.

5-(4-chlorophenyl)-2-(1H-pyrrol-2-yl)oxazole (**18**)

Compound **6K** (317 mg, 1.2 mmol), PPh_3 (1.26 g, 4.8 mmol), C_2Cl_6 (0.99 g, 4.5 mmol) and Et_3N (1.33 mL,

9.6 mmol) were mixed in DCM. Following the general method, compound **18** was got as yellow solid (152 mg) in a 52% yield. Melting point: 194 °C. ^{1}H NMR (400 MHz, DMSO-d_6) δ 11.96 (s, 1H), 7.81 (d, J = 8.2 Hz, 2H), 7.76 (s, 1H), 7.56 (d, J = 8.2 Hz, 2H), 7.02 (d, J = 3.0 Hz, 1H), 6.81 (dd, J = 3.9, 2.1 Hz, 1H), 6.23 (q, J = 2.7 Hz, 1H); ^{13}C NMR (100 MHz, DMSO-d_6) δ 155.35, 148.71, 133.10, 129.61, 126.39, 124.76, 122.63, 120.87, 112.22, 96.72; HRMS calcd for ($C_{13}H_9ClN_2O$ + H)$^+$ 244.0403, found 244.0401.

2-(4-bromo-1*H*-pyrrol-2-yl)-5-(4-chlorophenyl)oxazole (**19**)

Compound **6L** (367 mg, 1.1 mmol), PPh_3 (1.12 g, 4.28 mmol), C_2Cl_6 (0.88 g, 3.75 mmol) and Et_3N (1.18 mL, 8.56 mmol) were mixed in DCM. Following the general method, compound **19** was got as yellow solid (155 mg) in a 45% yield. Melting point: 239 °C. ^{1}H NMR (400 MHz, DMSO-d_6) δ 12.35 (s, 1H), 7.85 – 7.73 (m, 3H), 7.56 (d, J = 8.4 Hz, 2H), 7.18 (dd, J = 3.0, 1.6 Hz, 1H), 6.88 (dd, J = 2.7, 1.5 Hz, 1H); ^{13}C NMR (100 MHz, DMSO-d_6) δ 155.35, 148.71, 133.10, 129.61, 126.84, 125.94, 124.76, 122.63, 120.87, 112.22, 96.72; HRMS calcd for ($C_{13}H_8BrClN_2O$ + H)$^+$ 322.9509, found 322.9501.

5-(4-chlorophenyl)-2-(furan-2-yl)oxazole (**20**)

Compound **6M** (246 mg, 0.94 mmol), PPh_3 (1.12 g, 4.28 mmol), C_2Cl_6 (0.88 g, 3.75 mmol) and Et_3N (1.03 mL, 7.48 mmol) were mixed in DCM. Following the general method, compound **19** was got as yellow solid (92 mg) in a 40% yield. Melting point: 79 °C. ^{1}H NMR (400 MHz, DMSO-d_6) δ 7.97 (d, J = 1.8 Hz, 1H), 7.87 (s, 1H), 7.83 – 7.75 (m, 2H), 7.55 (d, J = 8.3 Hz, 2H), 7.26 (d, J = 3.5 Hz, 1H), 6.75 (dd, J = 3.6, 1.8 Hz, 1H); ^{13}C NMR (100 MHz, DMSO-d_6) δ 153.79, 149.61, 146.20, 142.38, 133.53, 129.67, 127.93, 125.84, 123.94; HRMS calcd for ($C_{13}H_8ClNO_2$ + H)$^+$ 246.0244, found 246.0238.

5-(4-chlorophenyl)-2-(thiophen-2-yl)oxazole (**21**)

Compound **6N** (300 mg, 1.07 mmol), PPh_3 (1.01 g, 4.28 mmol), C_2Cl_6 (0.88 g, 3.75 mmol) and Et_3N (1.18 mL, 8.56 mmol) were mixed in DCM. Following the general method, compound **21** was got as yellow solid (103 mg) in a 37% yield. Melting point: 98 °C. ^{1}H NMR (400 MHz, DMSO-d_6) δ 7.86 – 7.78 (m, 5H), 7.56 (d, J = 8.4 Hz, 2H), 7.25 (t, J = 4.4 Hz, 1H); ^{13}C NMR (100 MHz, DMSO-d_6) δ 157.27, 149.70, 133.46, 130.40, 129.67, 129.43, 128.71, 126.56, 126.13, 125.23; HRMS calcd for ($C_{13}H_8ClNOS$ + H)$^+$ 262.0015, found 262.0011.

5-(4-bromophenyl)-2-(1*H*-pyrrol-2-yl)oxazole (**22**)

Compound **6O** (244 mg, 0.79 mmol), PPh_3 (0.83 g, 3.16 mmol), C_2Cl_6 (0.65 g, 2.77 mmol) and Et_3N (0.89 mL, 6.32 mmol) were mixed in DCM. Following the general method, compound **22** was got as yellow

solid (118 mg) in a 52% yield. Melting point: 205 ^{o}C. ^{1}H NMR (400 MHz, DMSO-d$_6$) δ 11.96 (s, 1H), 7.88 – 7.51 (m, 5H), 7.03 (s, 1H), 6.84 (d, *J* = 20.4 Hz, 1H), 6.23 (d, *J* = 3.2 Hz, 1H); ^{13}C NMR (100 MHz, DMSO-*d*$_6$) *δ* 157.04, 150.71, 130.14, 129.55, 129.02, 128.58, 127.67, 124.46; HRMS calcd for $(C_{13}H_9BrN_2O + H)^+$ 287.9898, found 287.9893.

2-(4-bromo-1H-pyrrol-2-yl)-5-(4-bromophenyl)oxazole (**23**)

Compound **6P** (271 mg, 0.7 mmol), PPh_3 (0.73 g, 2.8 mmol), C_2Cl_6 (0.58 g, 2.45 mmol) and Et_3N (0.77 mL, 5.6 mmol) were mixed in DCM. Following the general method, compound **23** was got as yellow solid (108 mg) in a 42% yield. Melting point: >240 ^{o}C. ^{1}H NMR (400 MHz, DMSO-d$_6$) δ 12.35 (s, 1H), 7.81 (s, 1H), 7.76 (d, *J* = 8.6 Hz, 2H), 7.70 (d, *J* = 8.7 Hz, 2H), 7.18 (dd, *J* = 2.9, 1.6 Hz, 1H), 6.88 (t, *J* = 2.1 Hz, 1H); ^{13}C NMR (100 MHz, DMSO-d$_6$) δ 155.38, 148.76, 132.50, 127.17, 126.16, 124.86, 122.65, 121.68, 120.86, 112.24, 96.73; HRMS calcd for $(C_{13}H_8Br_2N_2O + H)^+$ 365.9003, found 365.9001.

5-(4-bromophenyl)-2-(furan-2-yl)oxazole (**24**)

Compound **6Q** (254 mg, 0.82 mmol), PPh_3 (0.86 g, 3.28 mmol), C_2Cl_6 (0.68 g, 2.87 mmol) and Et_3N (0.91 mL, 6.56 mmol) were mixed in DCM. Following the general method, compound **24** was got as yellow solid (107 mg) in a 45% yield. Melting point: 78 ^{o}C. ^{1}H NMR (400 MHz, DMSO-*d*$_6$) *δ* 7.98 (d, *J* = 1.6 Hz, 1H), 7.90 (s, 1H), 7.72 (q, *J* = 8.7 Hz, 4H), 7.28 (d, *J* = 3.5 Hz, 1H), 6.77 (dd, *J* = 3.5, 1.8 Hz, 1H); ^{13}C NMR (100 MHz, DMSO-*d*$_6$) *δ* 153.81, 149.66, 146.21, 142.38, 132.57, 126.59, 125.20, 122.12, 112.86； HRMS calcd for $(C_{13}H_8BrNO2 + H)^+$ 289.9738, found 289.9831.

5-(4-bromophenyl)-2-(thiophen-2-yl)oxazole (**25**)

Compound **6R** (356 mg, 1.1 mmol), PPh_3 (1.15 g, 4.4 mmol), C_2Cl_6 (0.91 g, 3.85 mmol) and Et_3N (1.22 mL, 8.8 mmol) were mixed in DCM. Following the general method, compound **25** was got as yellow solid (138 mg) in a 41% yield. Melting point: 107 ^{o}C. ^{1}H NMR (400 MHz, Chloroform-d) δ 7.78 (dd, *J* = 3.8, 1.2 Hz, 1H), 7.59 (s, 3H), 7.49 (dd, *J* = 5.0, 1.2 Hz, 2H), 7.43 (s, 1H), 7.17 (dd, *J* = 5.0, 3.8 Hz, 1H)； ^{13}C NMR (100 MHz, Chloroform-*d*) δ 149.86, 132.17, 129.77, 129.06, 127.39, 126.70, 125.61, 123.85, 122.35; HRMS calcd for $(C_{13}H_8BrNO\ S+ H)^+$ 305.9510, found 305.9502.

5-(4-methoxyphenyl)-2-(1*H*-pyrrol-2-yl)oxazole (**26**)

Compound **6S** (259 mg, 1 mmol), PPh_3 (1.05 g, 4 mmol), C_2Cl_6 (0.83 g, 3.5 mmol) and Et_3N (1.1 mL, 8 mmol) were mixed in DCM. Following the general method, compound **26** was got as yellow solid (96 mg) in a 40% yield. Melting point: 166 ^{o}C. ^{1}H NMR (400 MHz, DMSO-*d*$_6$) *δ* 11.91 (s, 1H), 7.71 (d, *J* = 8.7 Hz, 2H),

7.55 (s, 1H), 7.11 – 6.97 (m, 3H), 6.77 (p, *J* = 1.8 Hz, 1H), 6.22 (q, *J* = 2.6 Hz, 1H), 3.81 (s, 3H); ^{13}C NMR (100 MHz, DMSO-*d*$_6$) *δ* 159.62, 155.87, 149.30, 125.71, 122.29, 120.90, 120.18, 114.99, 110.13, 55.73; HRMS calcd for $(C_{14}H_{12}N_2O_2 + H)^+$ 241.0899, found 241.0893.

2-(4-bromo-1*H*-pyrrol-2-yl)-5-(4-methoxyphenyl)oxazole (**27**)

Compound **6T** (283 mg, 0.84 mmol), PPh_3 (0.88 g, 3.36 mmol), C_2Cl_6 (0.69 g, 2.94 mmol) and Et_3N (0.93 mL, 6.72 mmol) were mixed in DCM. Following the general method, compound **27** was got as yellow solid (107 mg) in a 40% yield. Melting point: 209 ^{o}C. ^{1}H NMR (400 MHz, DMSO-*d*$_6$) *δ* 12.29 (s, 1H), 7.79 – 7.67 (m, 2H), 7.59 (s, 1H), 7.15 (dd, *J* = 2.9, 1.6 Hz, 1H), 7.10 – 7.01 (m, 2H), 6.83 (dd, *J* = 2.6, 1.6 Hz, 1H), 3.81 (s, 3H); ^{13}C NMR (100 MHz, DMSO-*d*$_6$) *δ* 159.80, 154.51, 149.86, 125.89, 124.12, 118.39, 115.01, 111.66, 96.58, 55.75; HRMS calcd for $(C_{14}H_{11}BrN_2O_2 + H)^+$ 319.1580, found 319.1573.

2-(furan-2-yl)-5-(4-methoxyphenyl)oxazole (**28**)

Compound **6U** (286 mg, 1.1 mmol), PPh_3 (1.15 g, 4.4 mmol), C_2Cl_6 (0.91 g, 3.85 mmol) and Et_3N (1.22 mL, 8.8 mmol) were mixed in DCM. Following the general method, compound **28** was got as yellow solid (97 mg) in a 36% yield. Melting point: 61 ^{o}C. ^{1}H NMR (400 MHz, DMSO-d$_6$) δ 7.94 (d, *J* = 1.7 Hz, 1H), 7.76 – 7.69 (m, 2H), 7.67 (s, 1H), 7.25 – 7.17 (m, 1H), 7.11 – 7.01 (m, 2H), 6.74 (dd, *J* = 3.5, 1.8 Hz, 1H), 3.81 (s, 3H); ^{13}C NMR (100 MHz, DMSO-d$_6$) δ 160.07, 153.00, 150.78, 145.83, 142.64, 126.12, 122.73, 120.27, 115.08, 112.79, 112.08, 55.75; HRMS calcd for $(C_{14}H_{11}NO_3 + H)^+$ 242.0739, found 242.0731.

5-(4-methoxyphenyl)-2-(thiophen-2-yl)oxazole (**29**)

Compound **6V** (283 mg, 1.03 mmol), PPh_3 (1.08 g, 4.12 mmol), C_2Cl_6 (0.85 g, 3.6 mmol) and Et_3N (1.14 mL, 8.24 mmol) were mixed in DCM. Following the general method, compound **29** was got as yellow solid (100 mg) in a 38% yield. Melting point: 89 ^{o}C. ^{1}H NMR (400 MHz, DMSO-d$_6$) δ 7.78 (d, *J* = 3.8 Hz, 1H), 7.76 – 7.69 (m, 1H), 7.63 (s, 4H), 7.30 – 7.19 (m, 1H), 7.13 – 7.00 (m, 1H), 3.81 (s, 3H); ^{13}C NMR (100 MHz, DMSO-d$_6$) δ 160.04, 156.39, 150.86, 129.76, 128.96, 128.23, 126.09, 122.83, 120.36, 115.08, 55.76; HRMS calcd for $(C_{14}H_{11}NOS + H)^+$ 258.0510, found 258.0503.

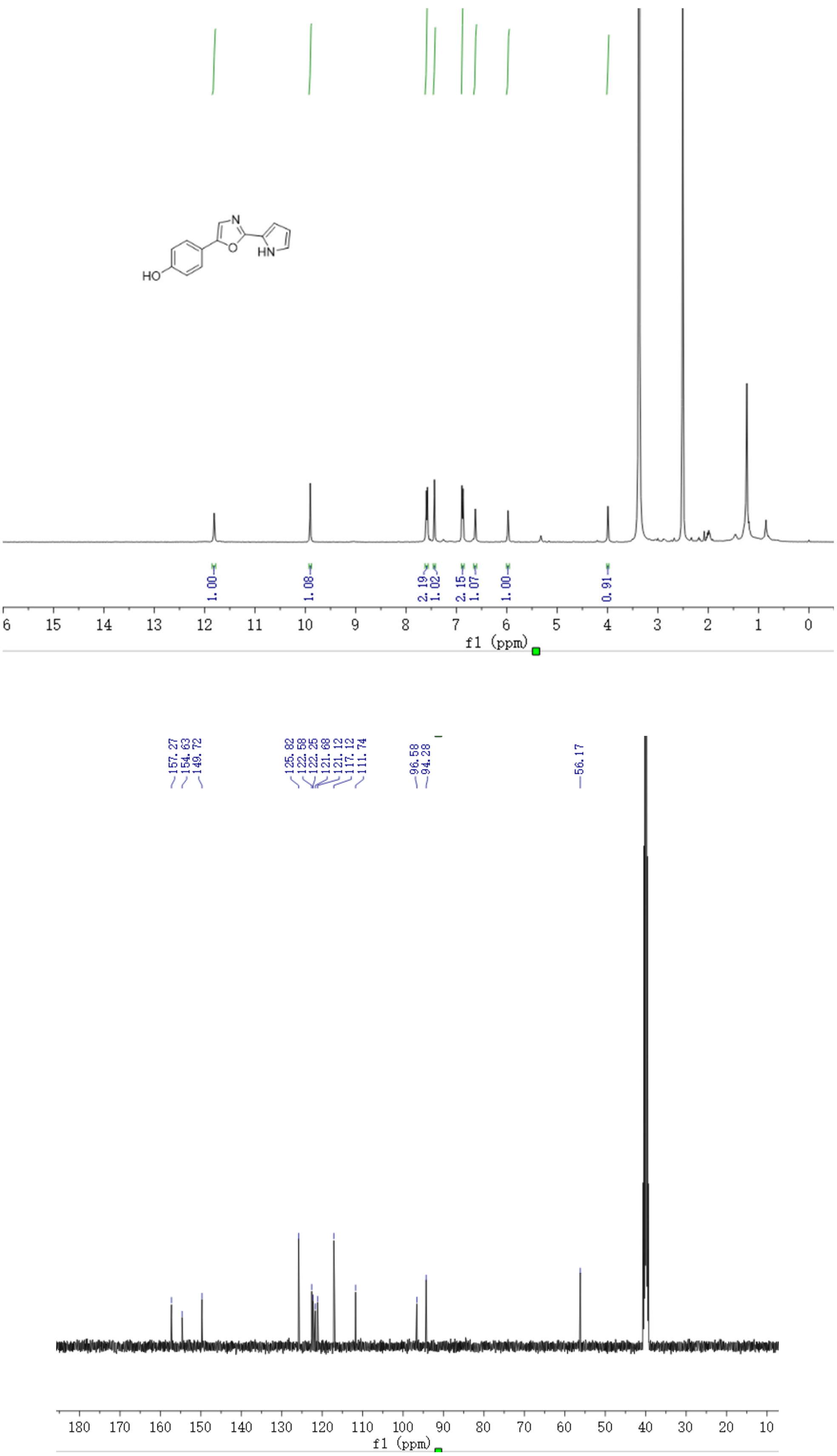

Figure S1. ^{1}H and ^{13}C NMR Spectrum of **1** in DMSO-d_6

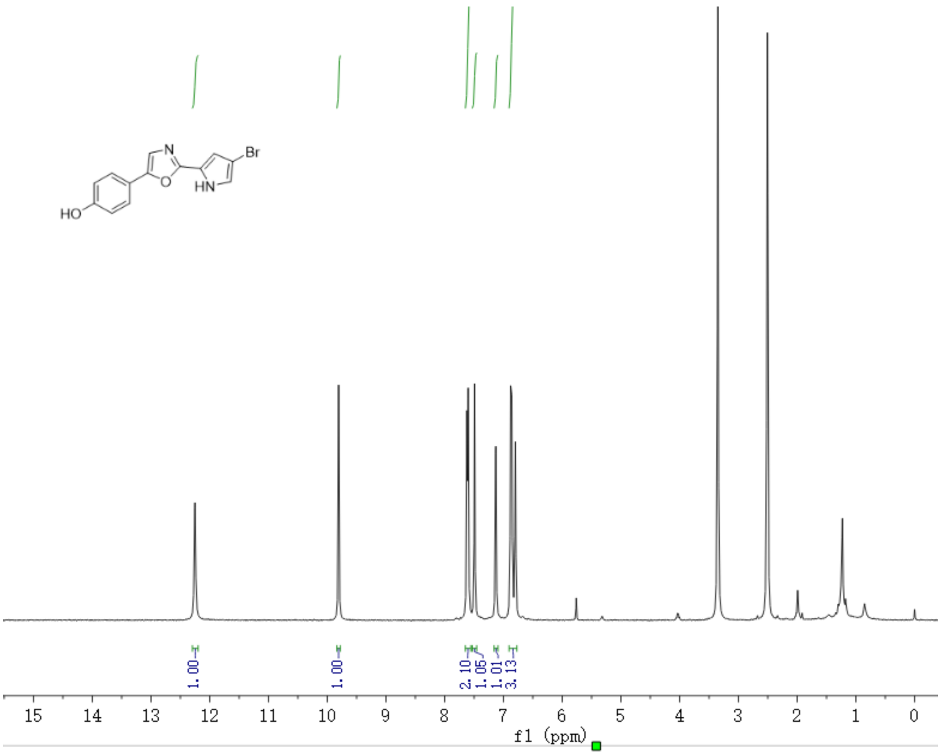

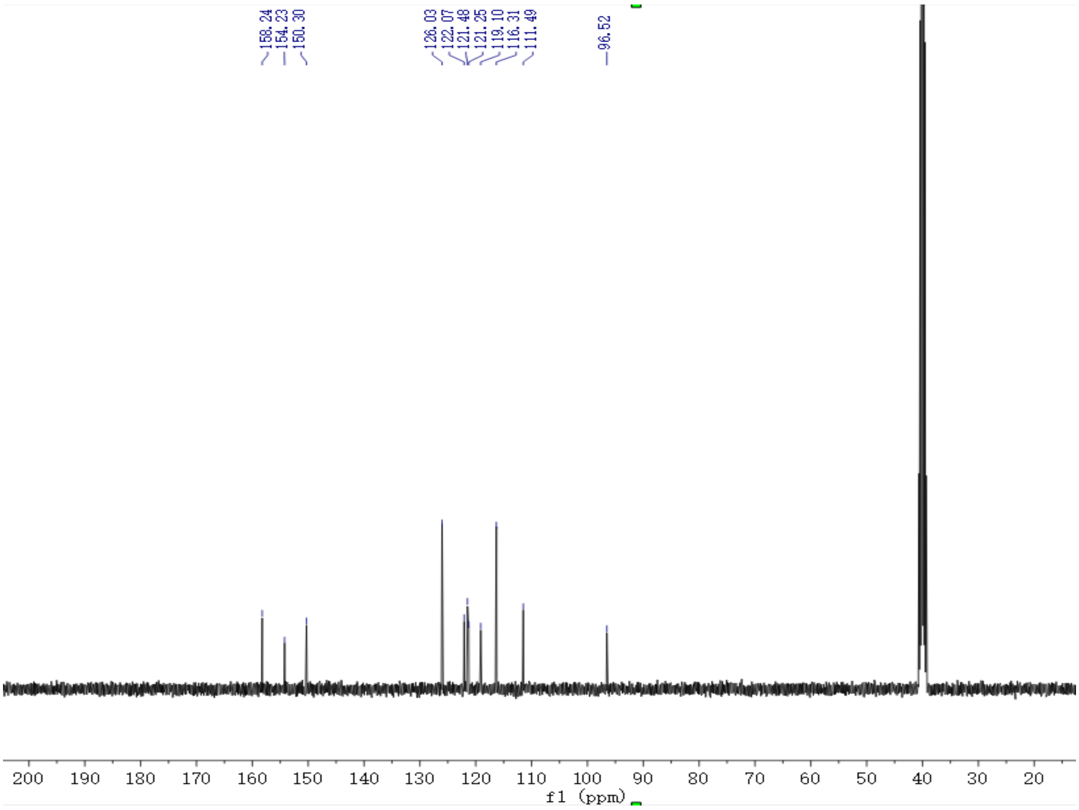

Figure S2. ^{1}H and ^{13}C NMR Spectrum of **2** in DMSO-*d*$_{6}$

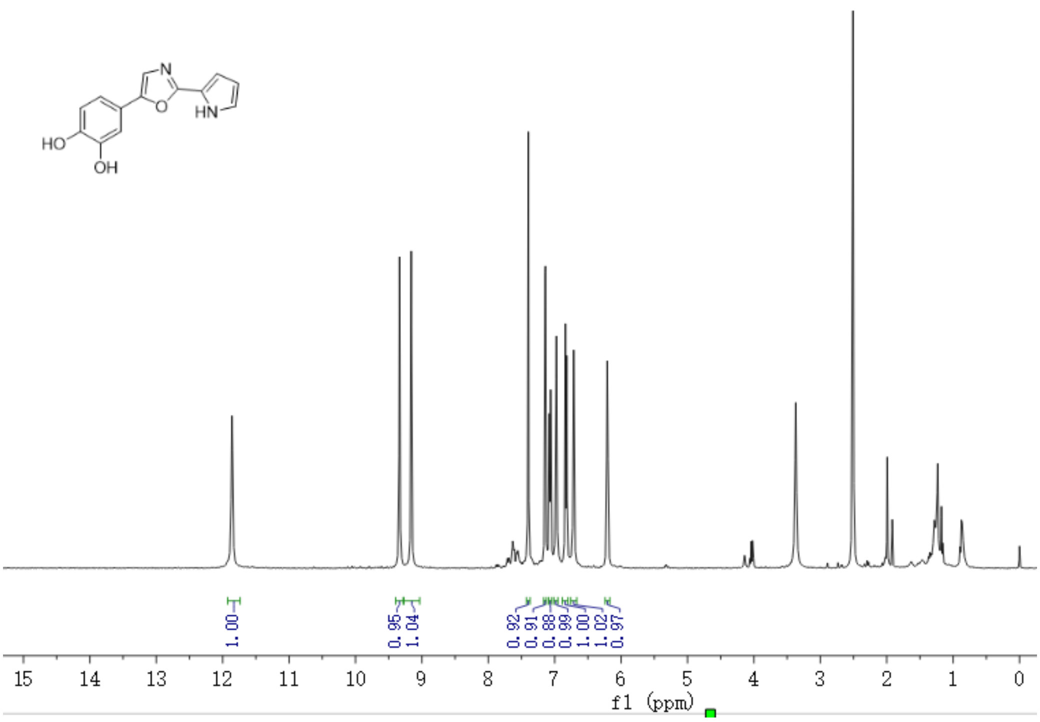

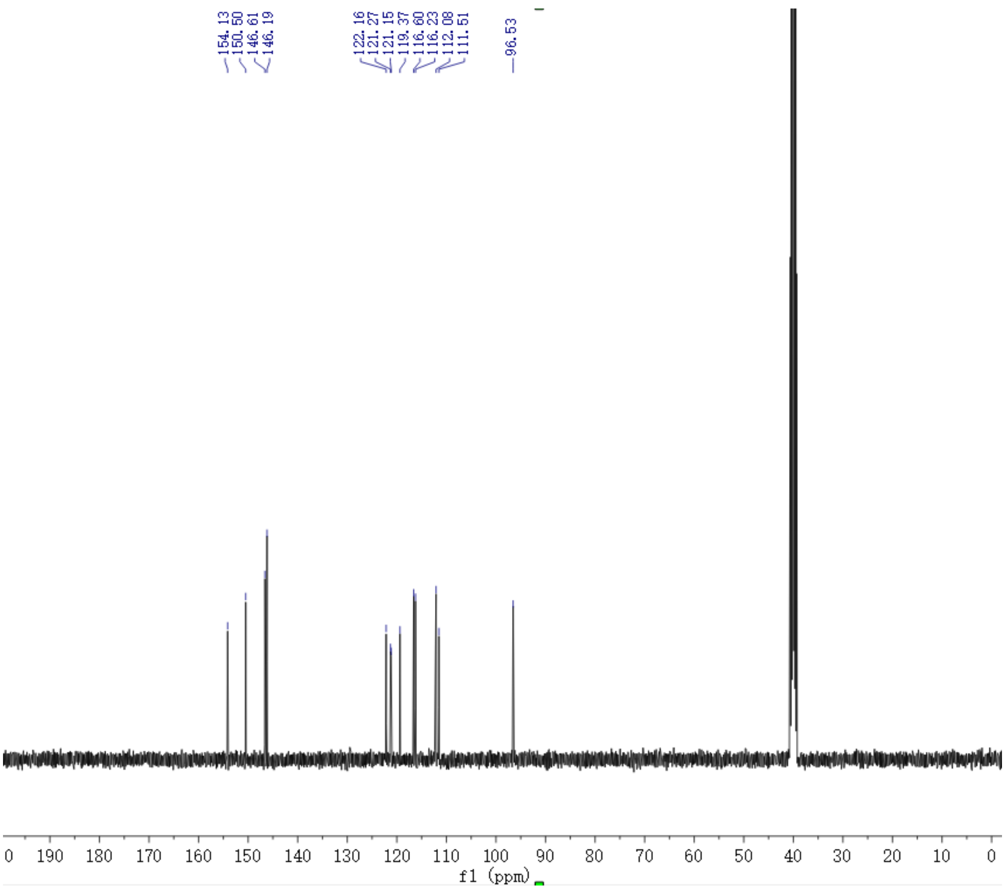

Figure S3. ^{1}H and ^{13}C NMR Spectrum of **3** in DMSO-*d*$_6$

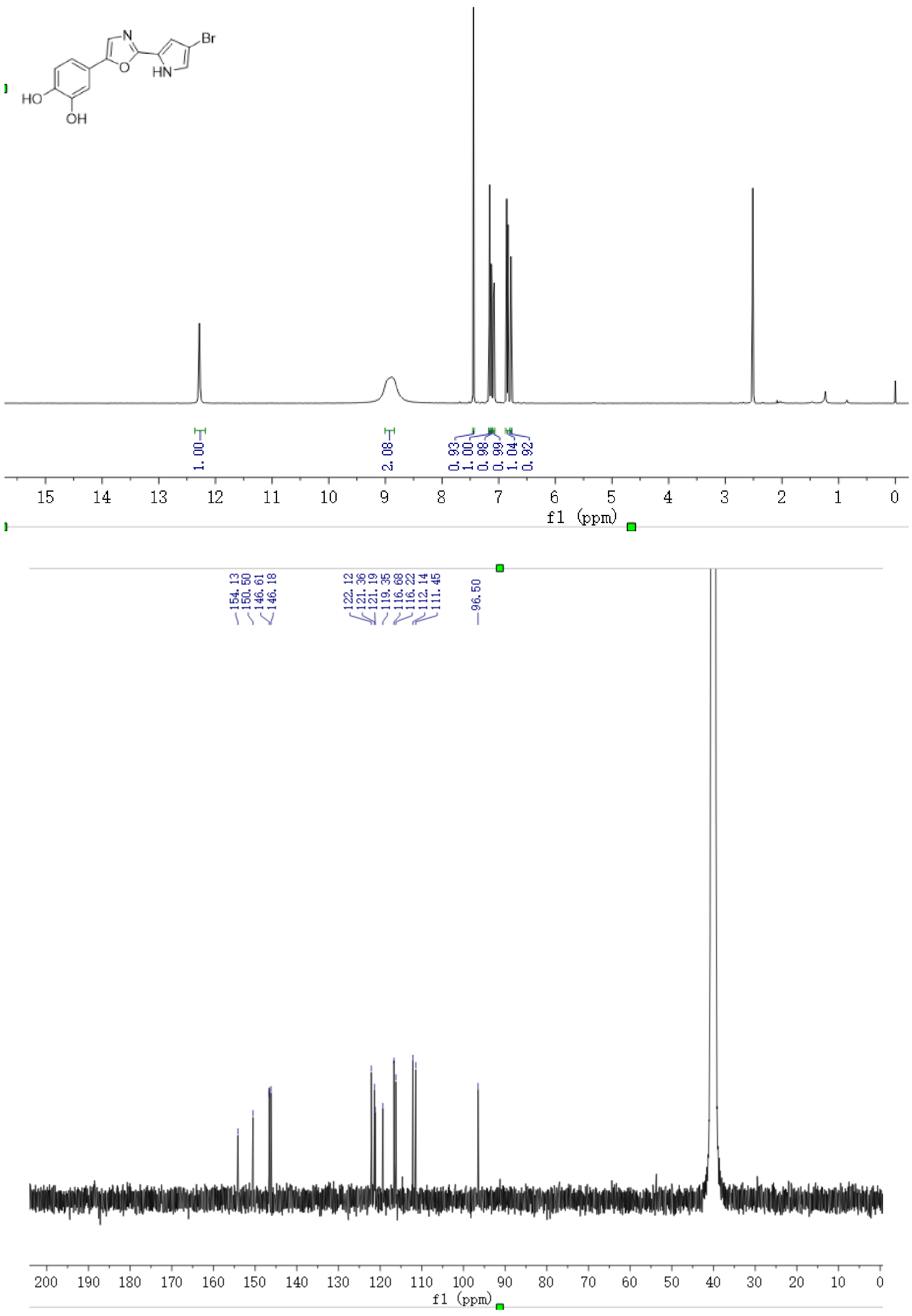

Figure S4. ^{1}H and ^{13}C NMR Spectrum of **4** in DMSO-d_6

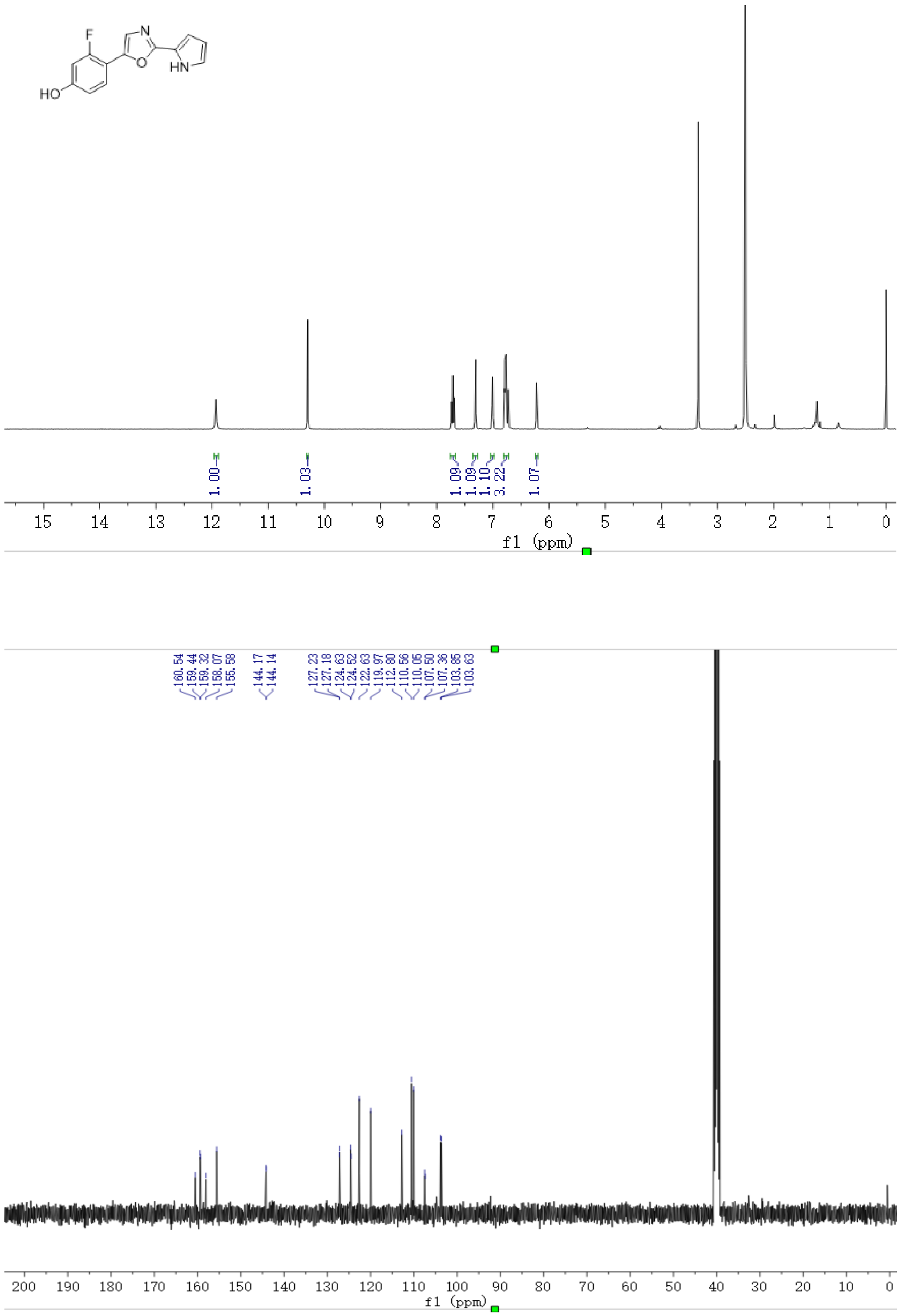

Figure S5. ^{1}H and ^{13}C NMR Spectrum of **5** in DMSO-d_6

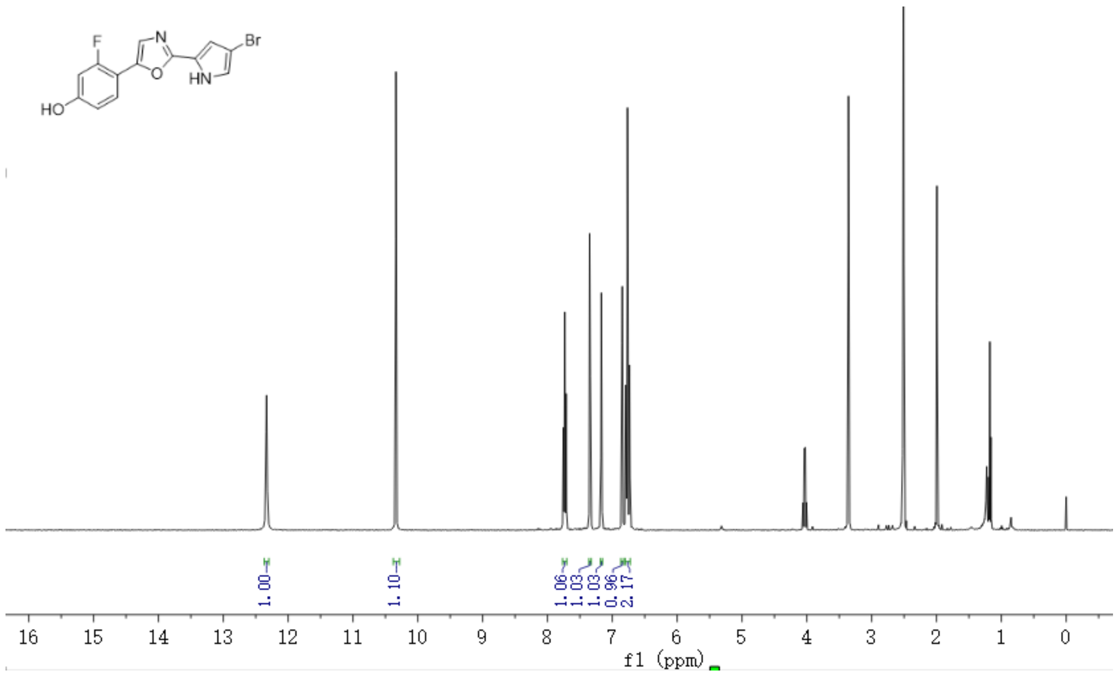

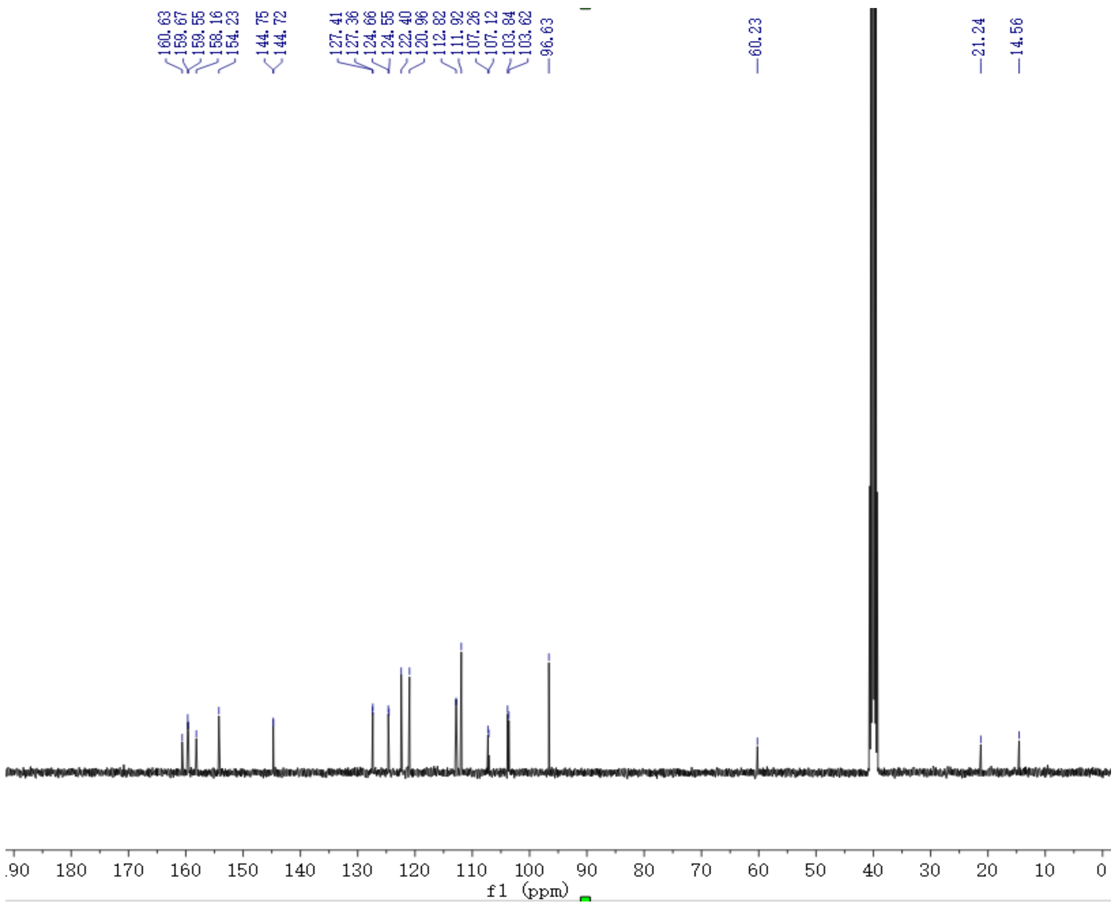

Figure S6. ^{1}H and ^{13}C NMR Spectrum of **6** in DMSO-d_6

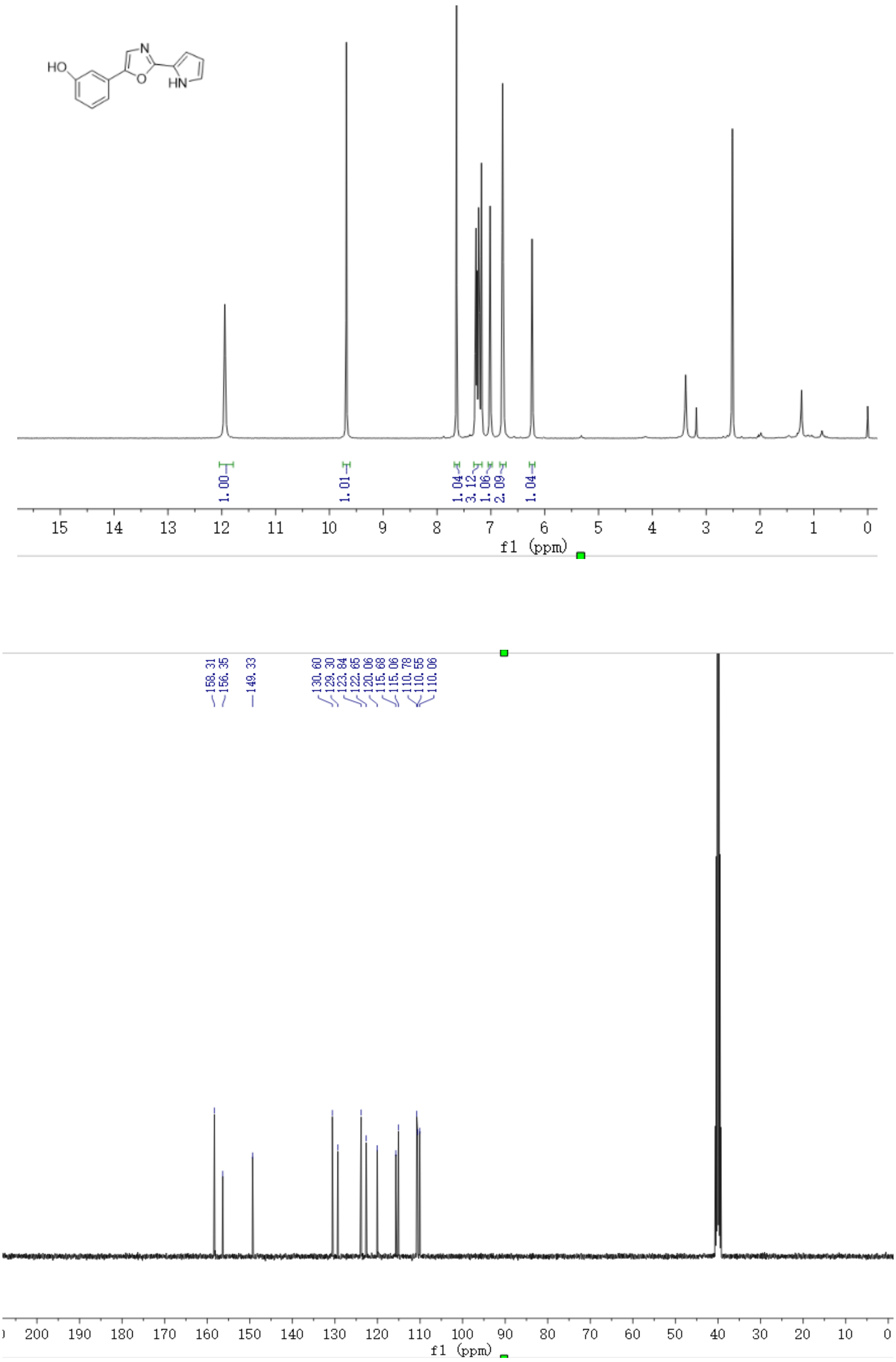

Figure S7. ^{1}H and ^{13}C NMR Spectrum of **7** in DMSO-d_6

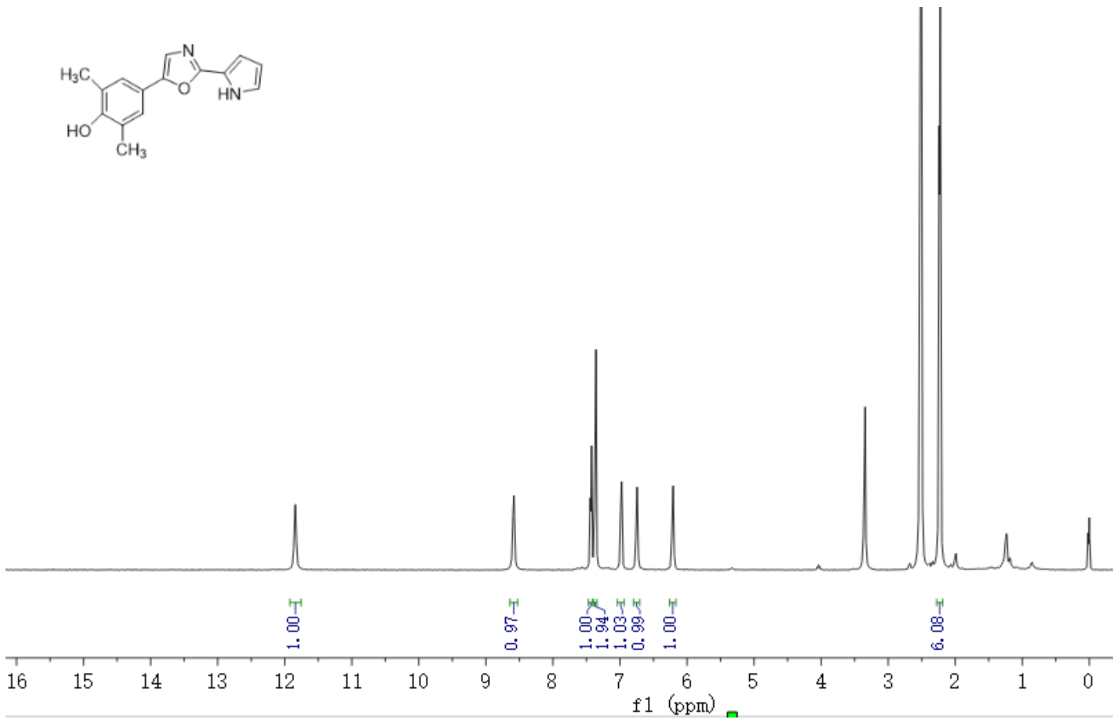

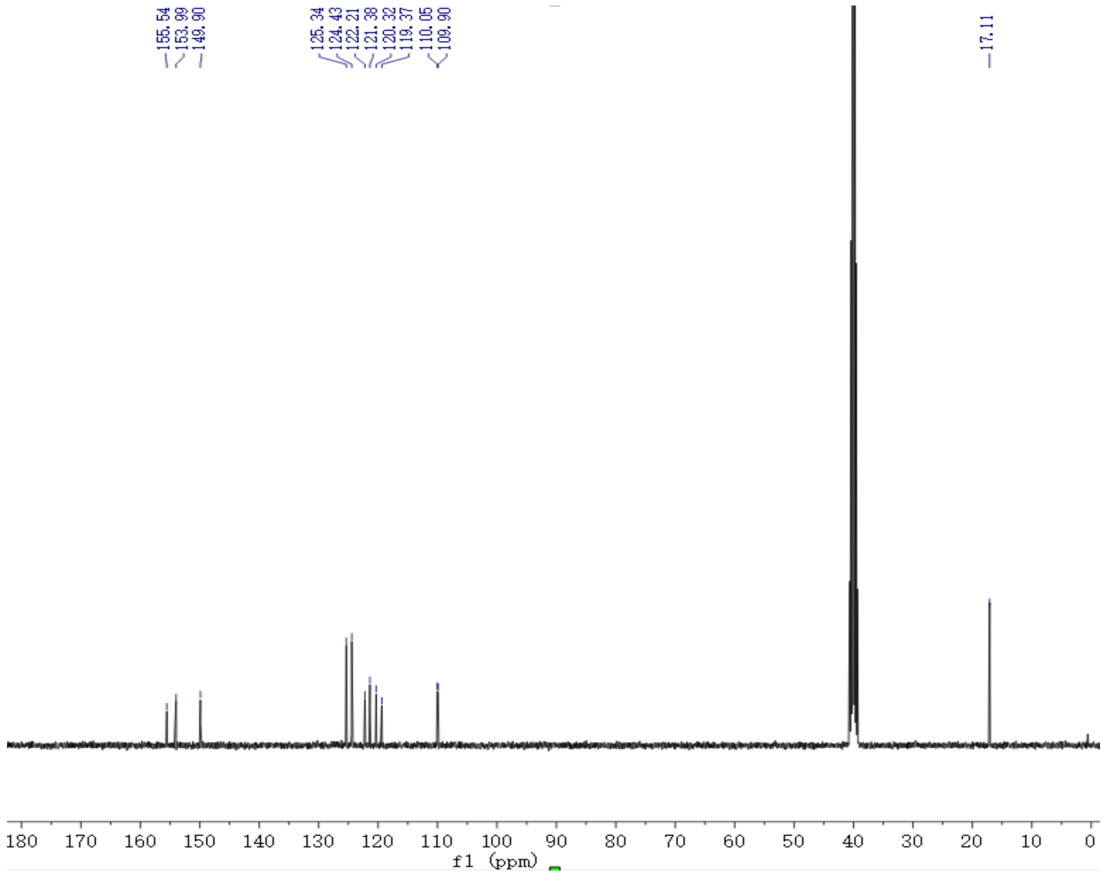

Figure S8. ^{1}H and ^{13}C NMR Spectrum of **8** in DMSO-*d*$_6$

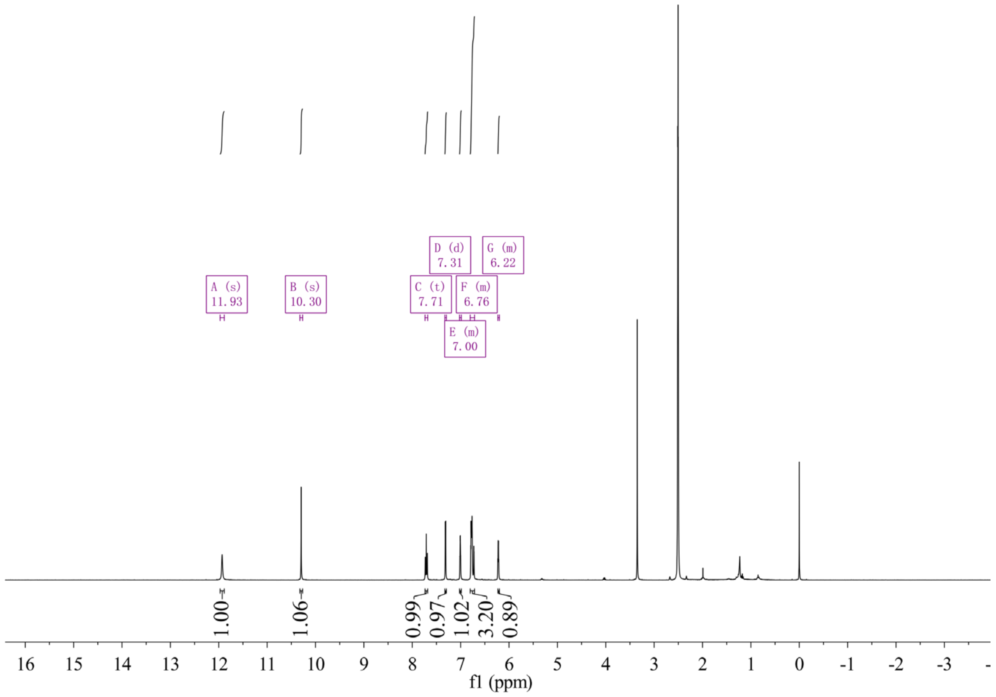

Figure S9. ^{1}H NMR Spectrum of **9** in DMSO-d_6

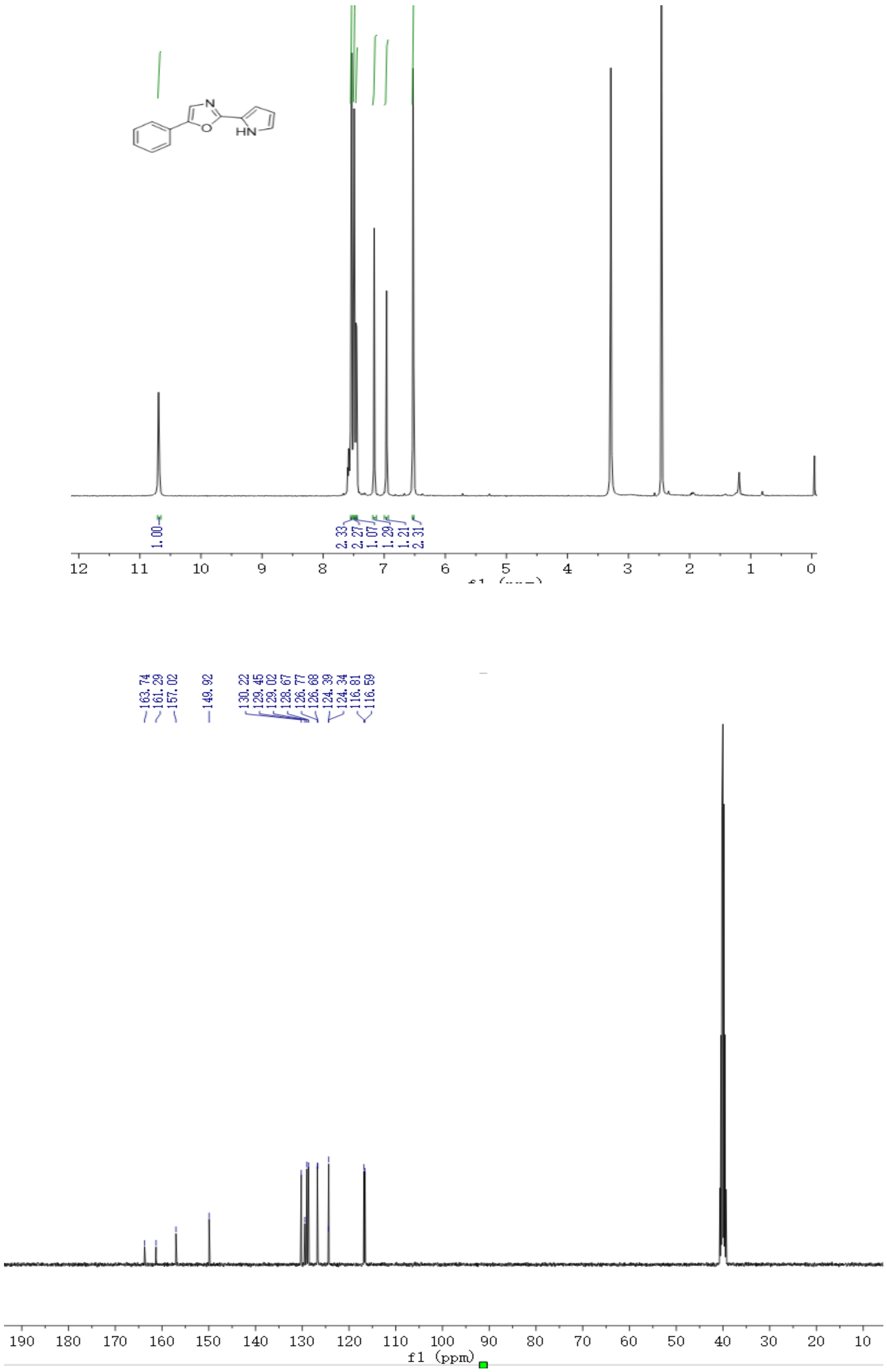

Figure S10. ^{1}H and ^{13}C NMR Spectrum of **10** in DMSO-d_6

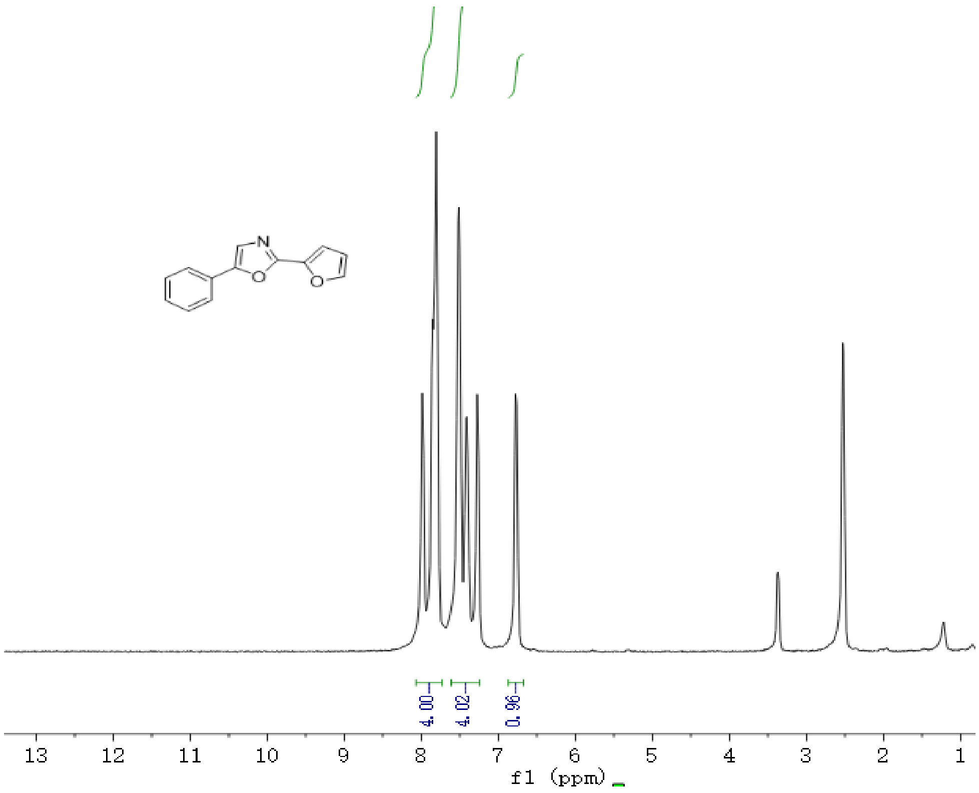

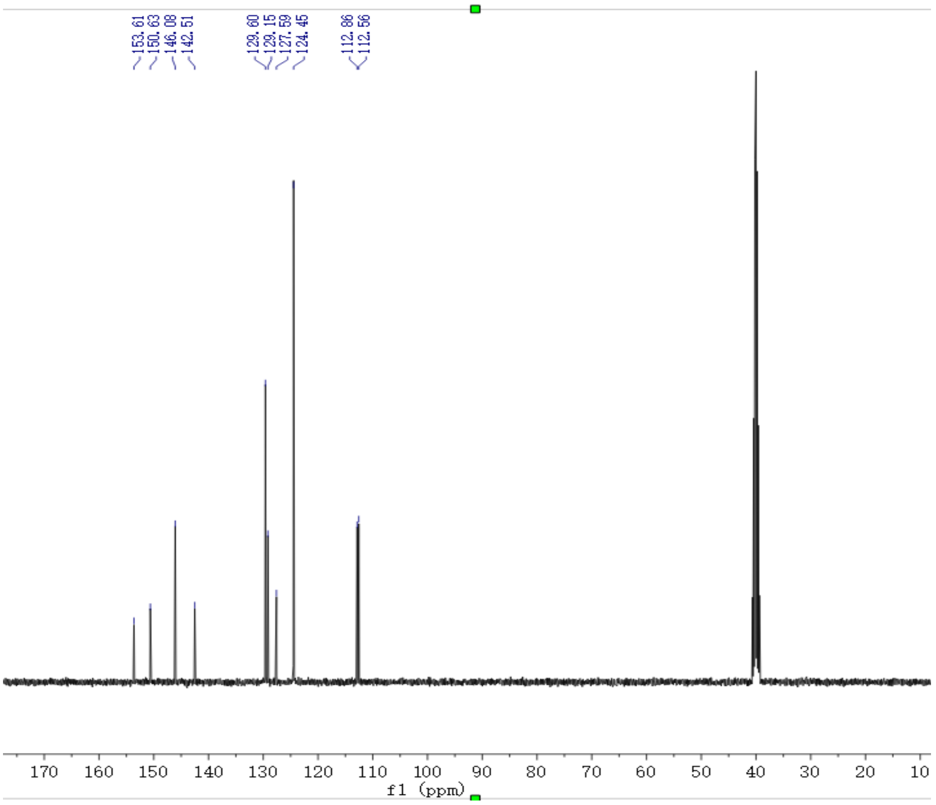

Figure S11. ^{1}H and ^{13}C Spectrum of **12** in DMSO-*d*$_6$

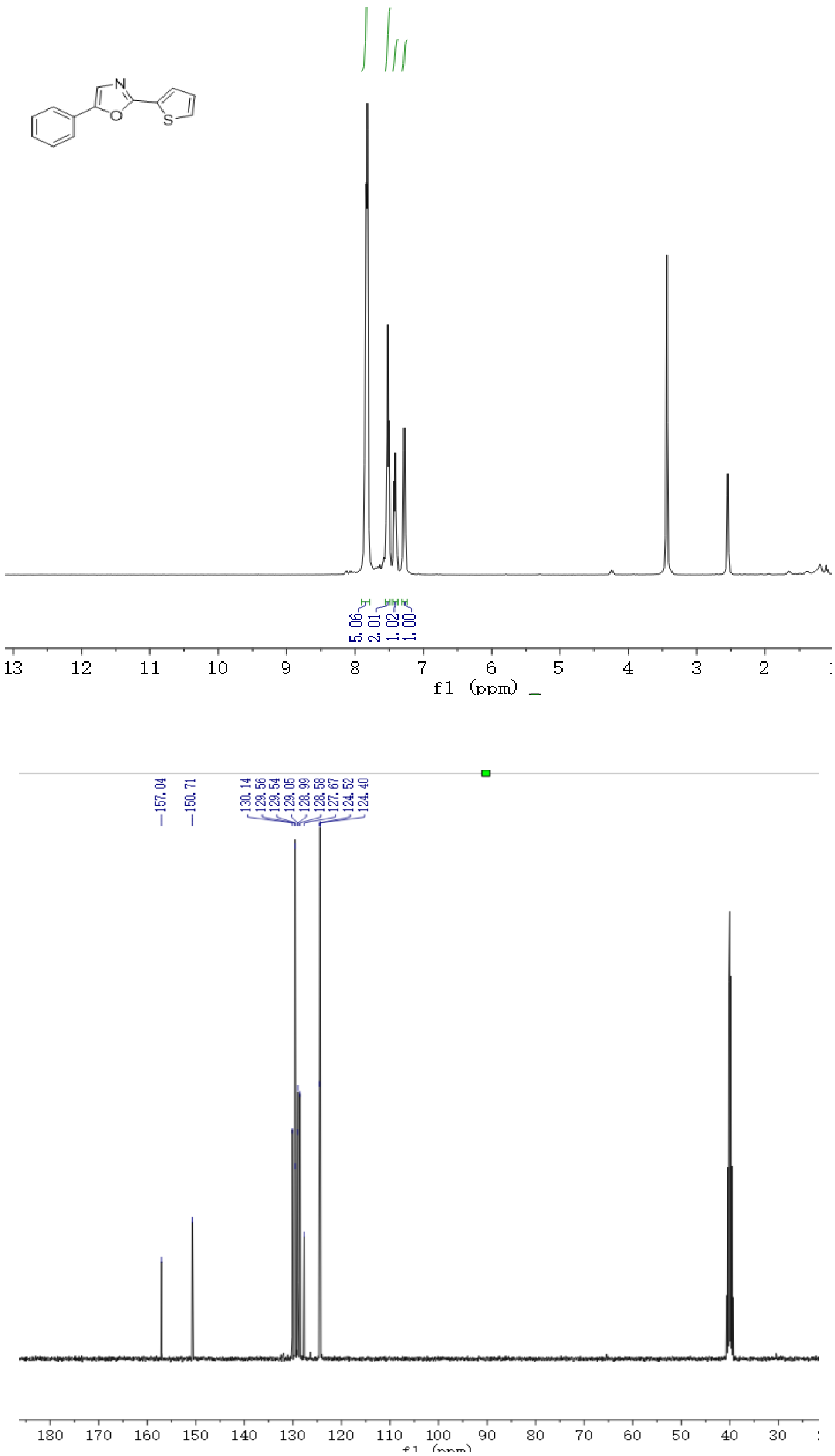

Figure S12. ^1H and ^{13}C Spectrum of **13** in DMSO-d_6

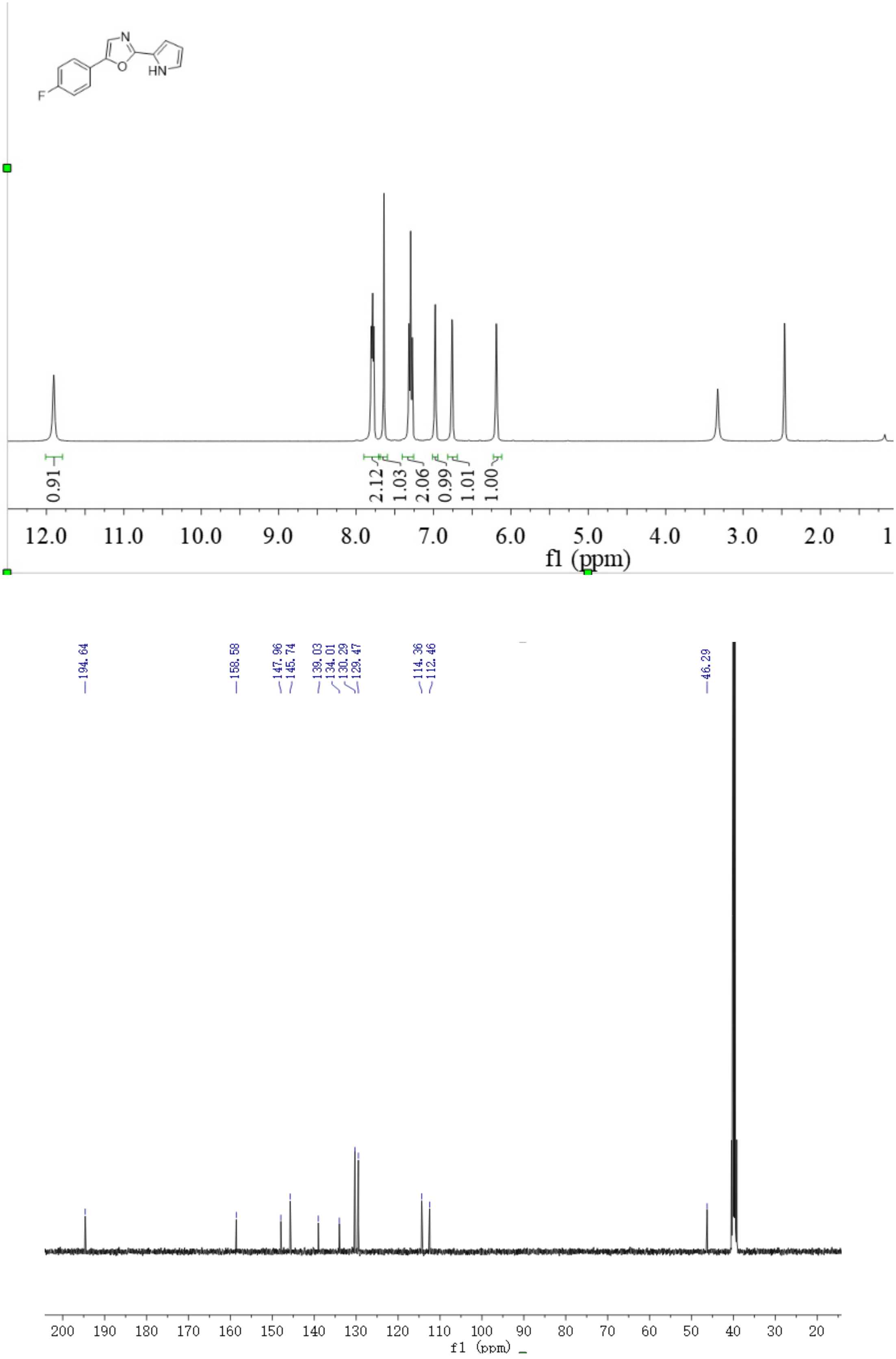

Figure S13. ^{1}H and [13]C Spectrum of **14** in DMSO-d_6

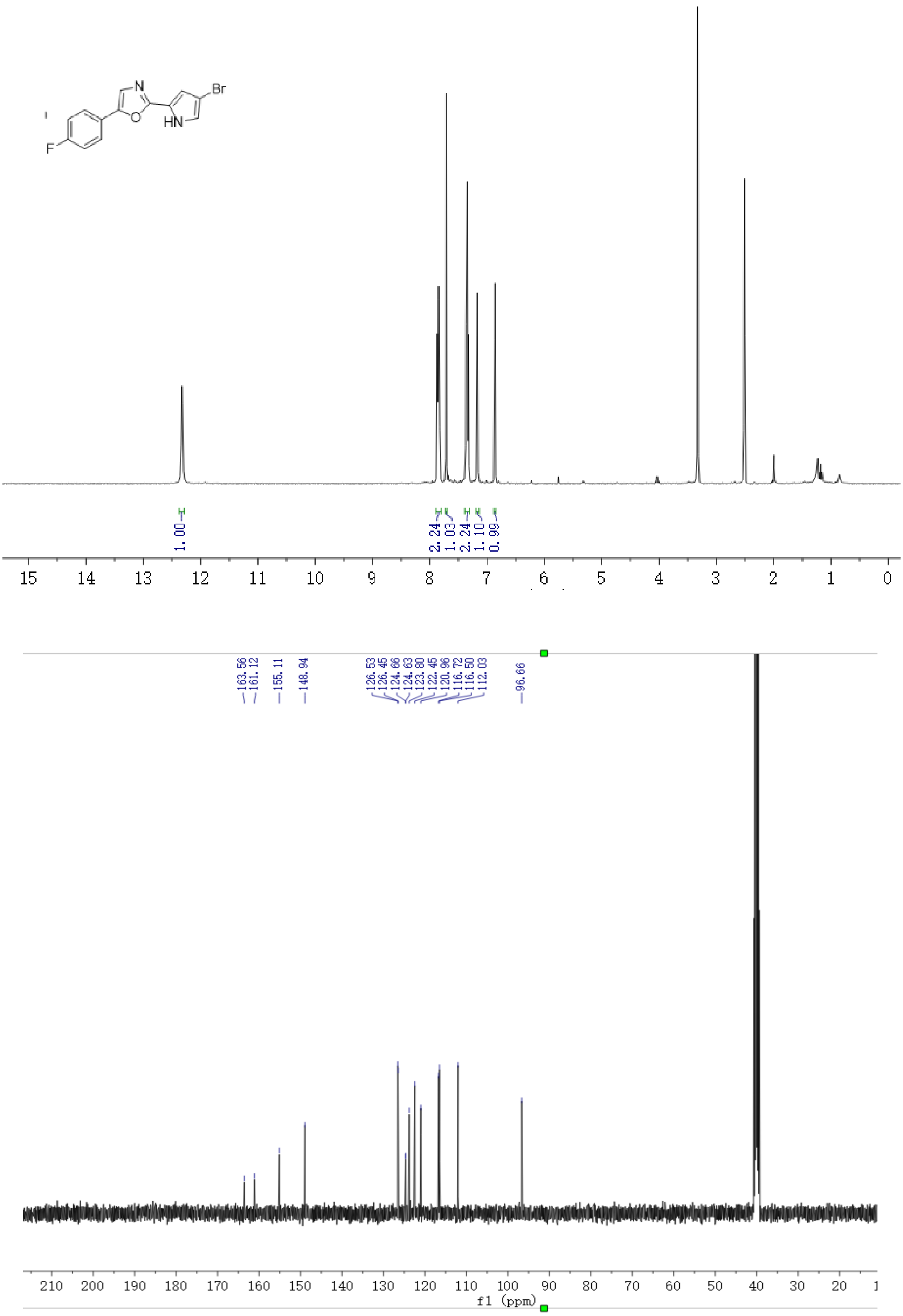

Figure S14. ^{1}H and ^{13}C Spectrum of **15** in DMSO-d_6

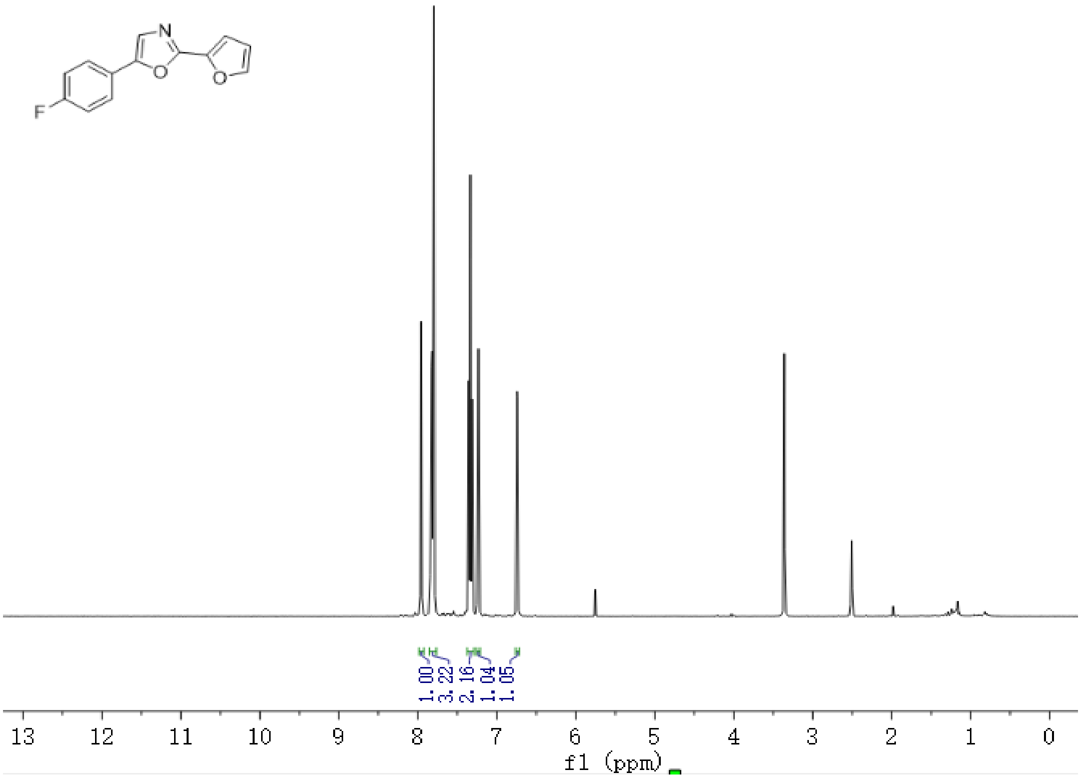

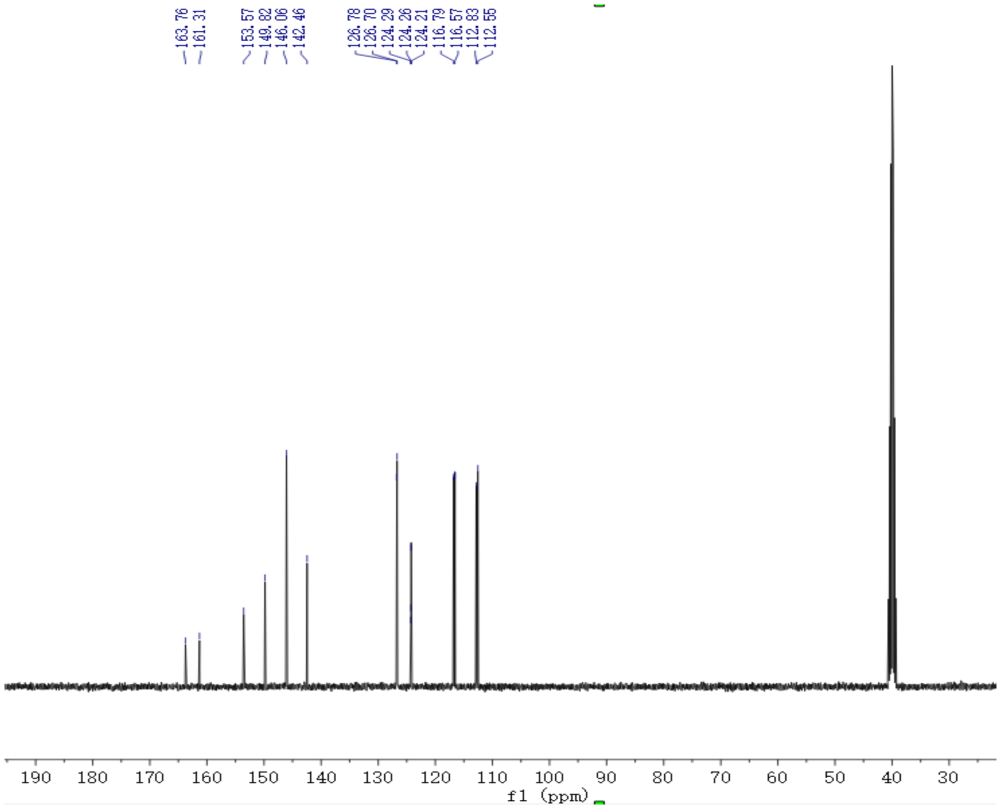

Figure S15. ^{1}H and ^{13}C Spectrum of **16** in DMSO-d_6

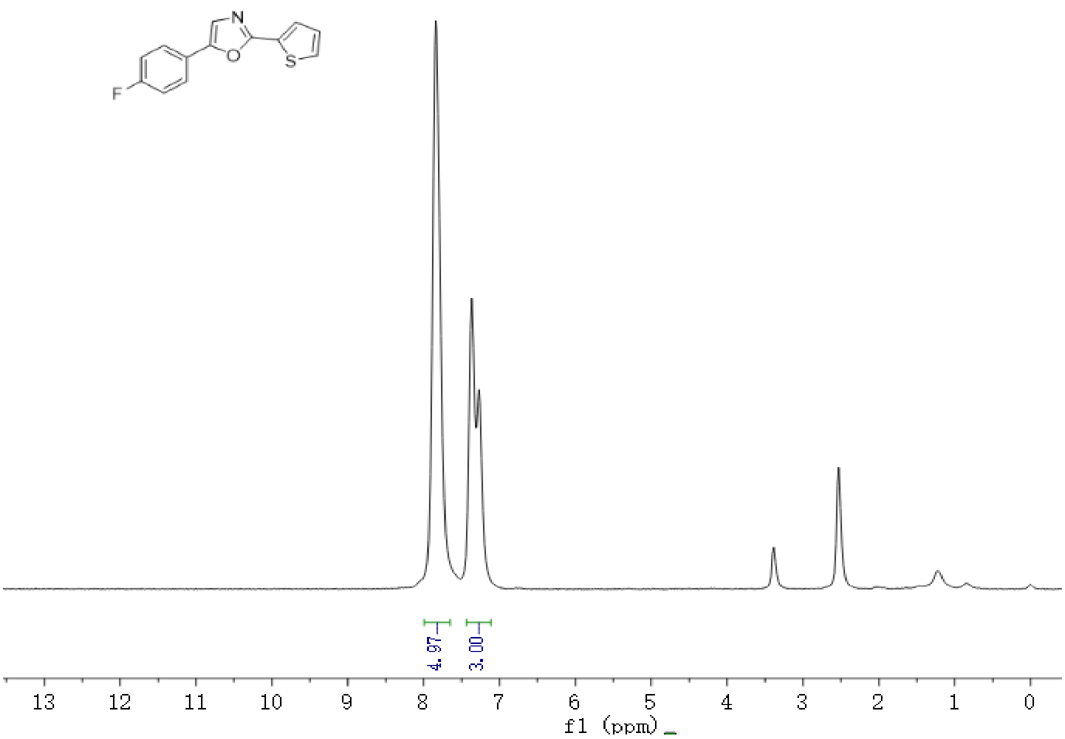

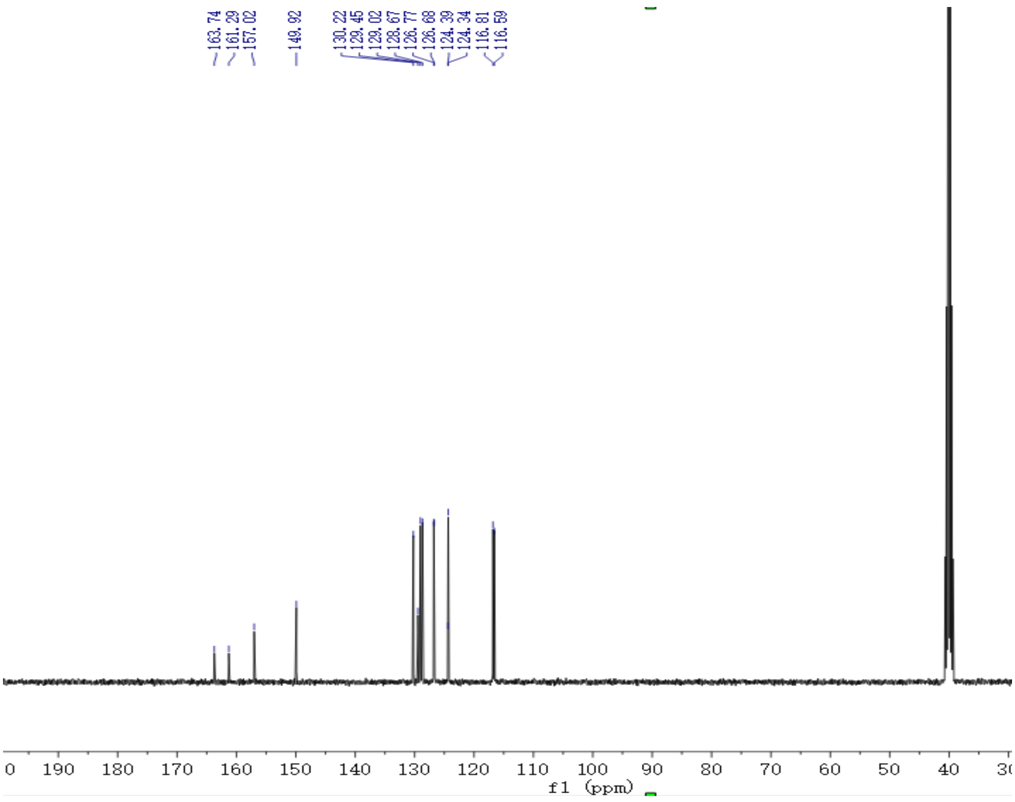

Figure S16. ^{1}H and ^{13}C Spectrum of **17** in DMSO-d_6

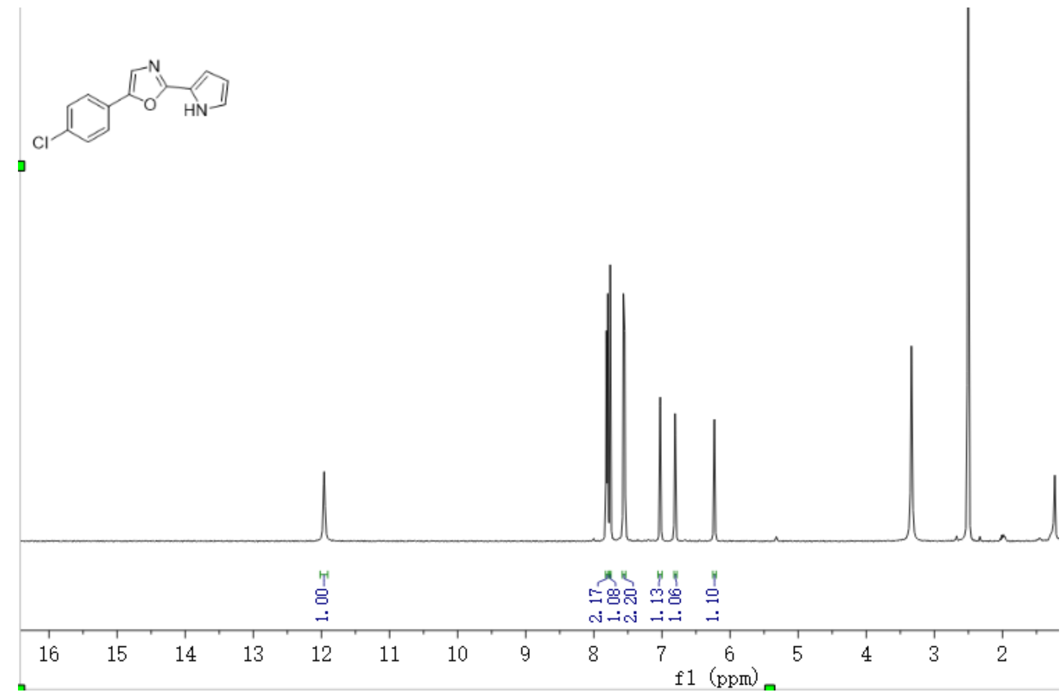

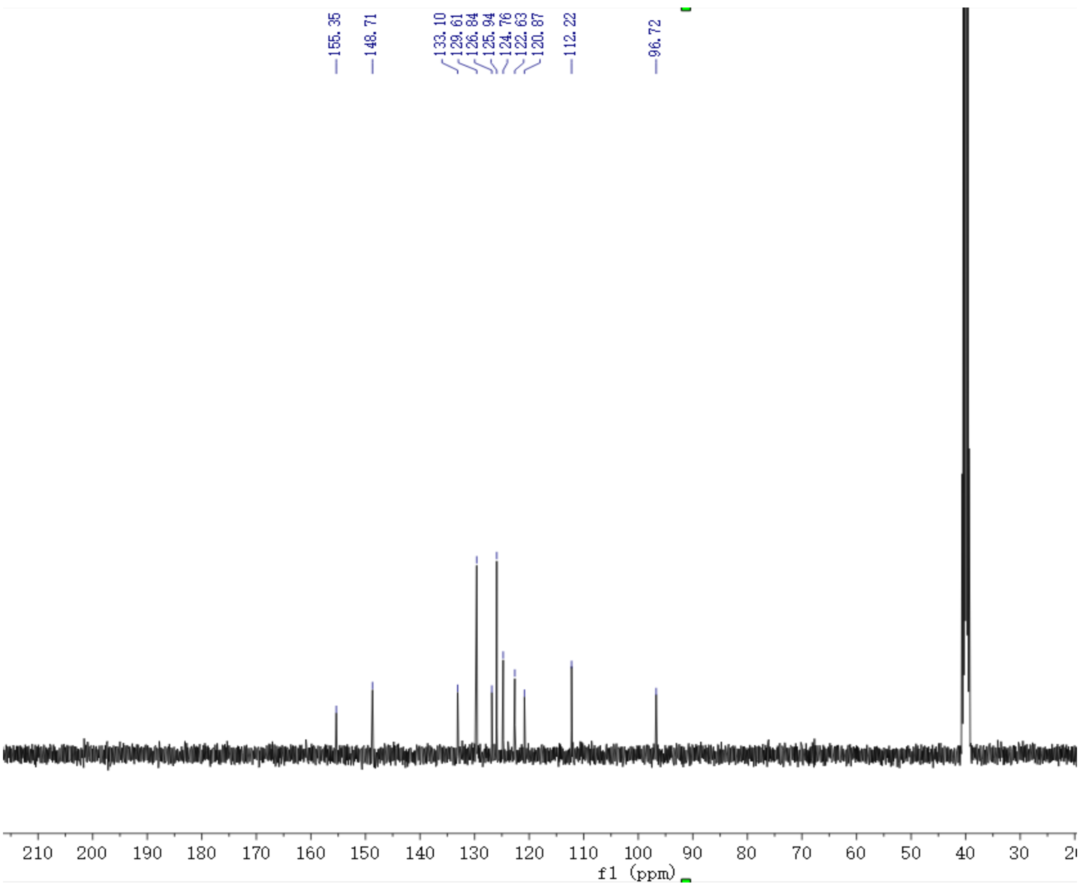

Figure S17. ^{1}H and ^{13}C Spectrum of **18** in DMSO-d_6

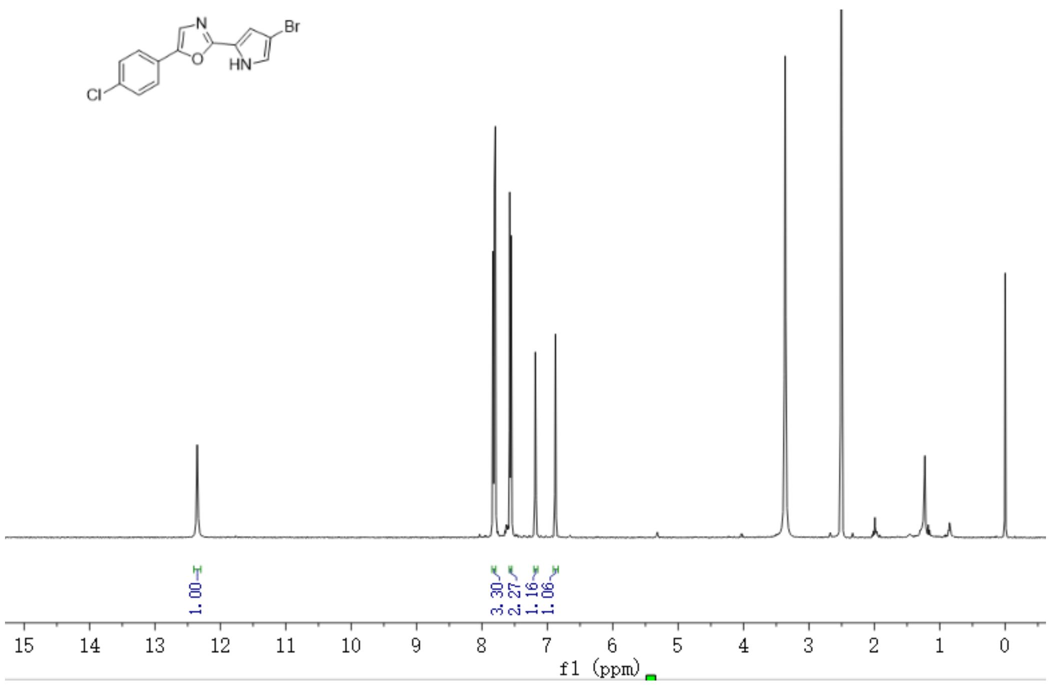

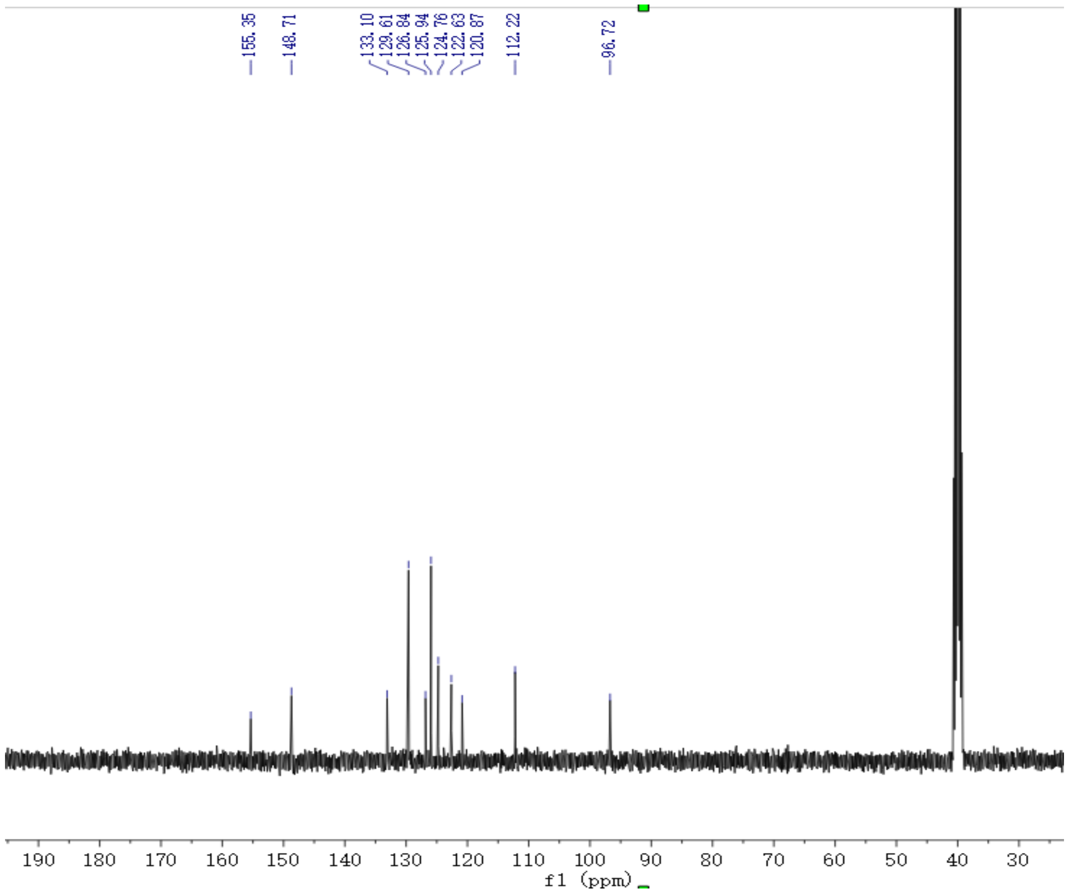

Figure S18. ^{1}H and ^{13}C Spectrum of **19** in DMSO-*d*$_6$

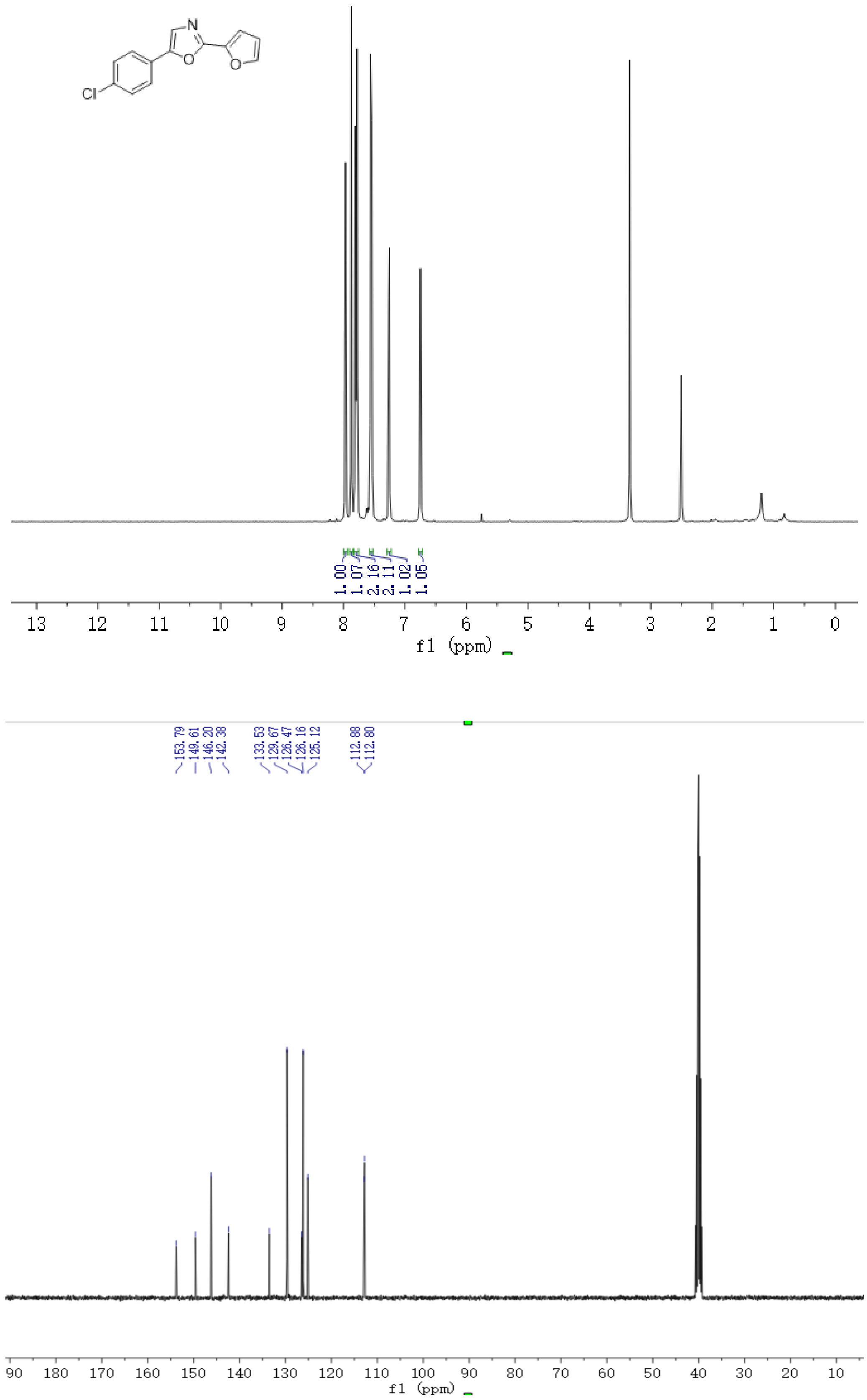

Figure S19. ^{1}H and ^{13}C Spectrum of **20** in DMSO-d_6

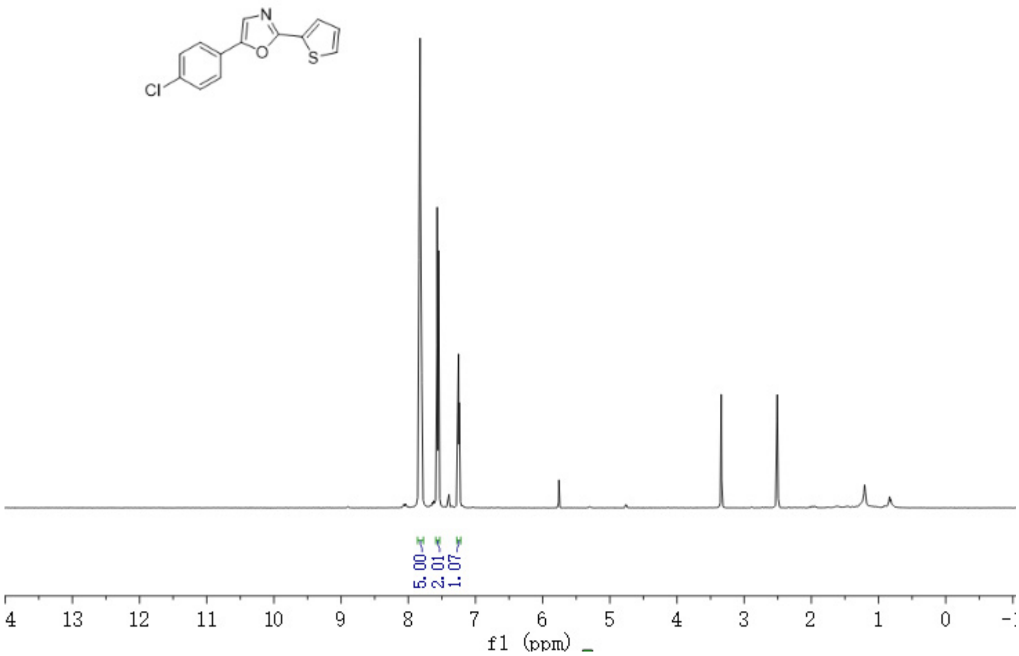

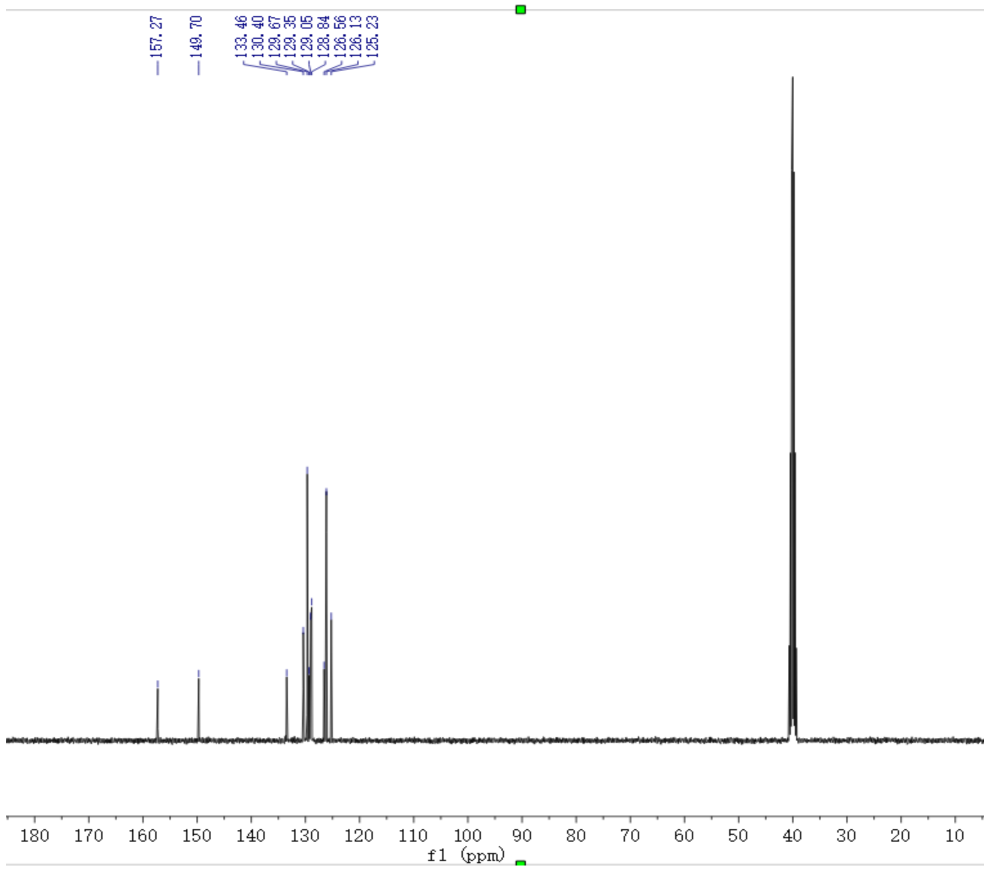

Figure S20. ^{1}H and ^{13}C Spectrum of **21** in DMSO-d_6

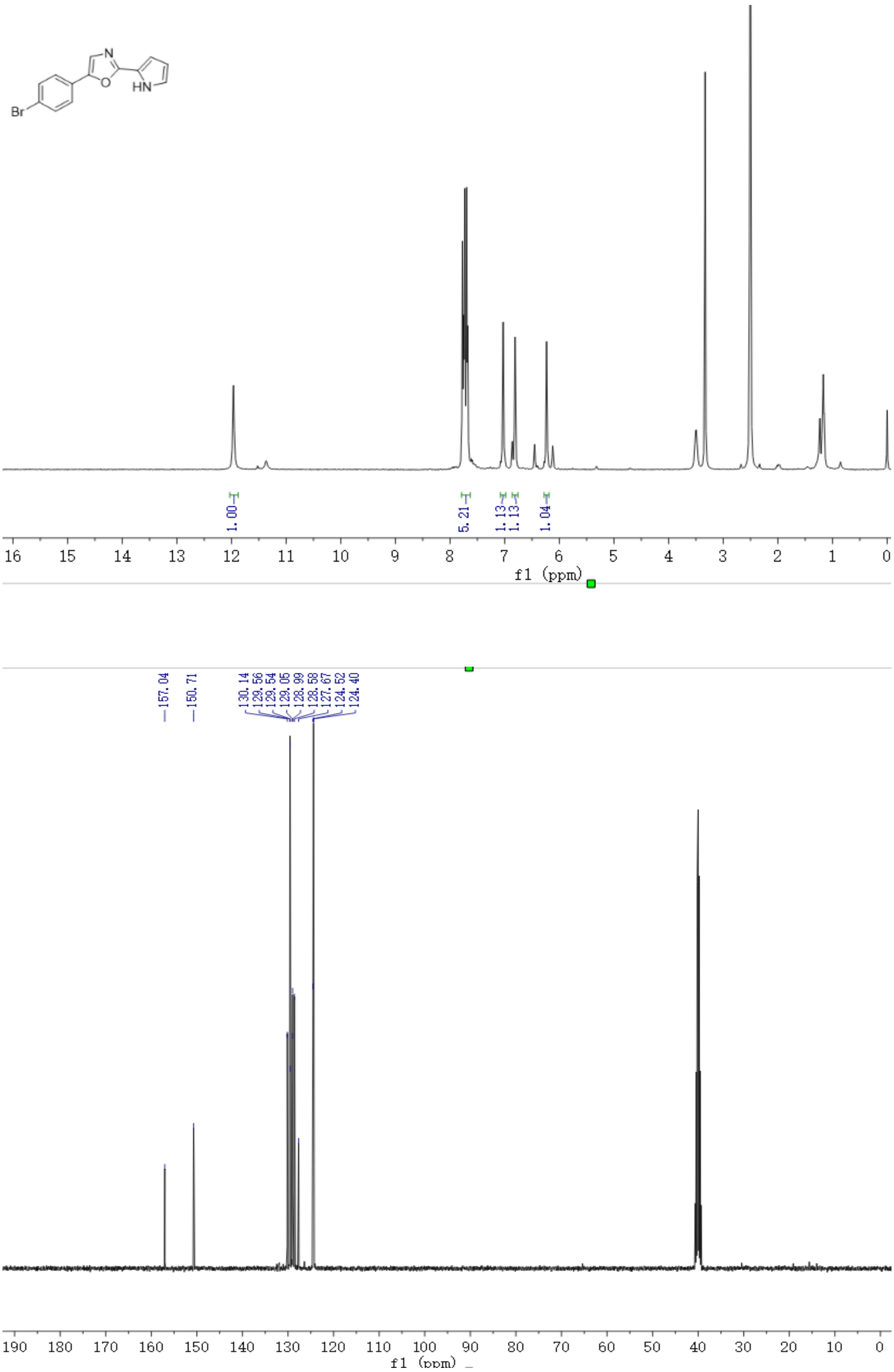

Figure S21. ^{1}H and ^{13}C Spectrum of **22** in DMSO-d_6

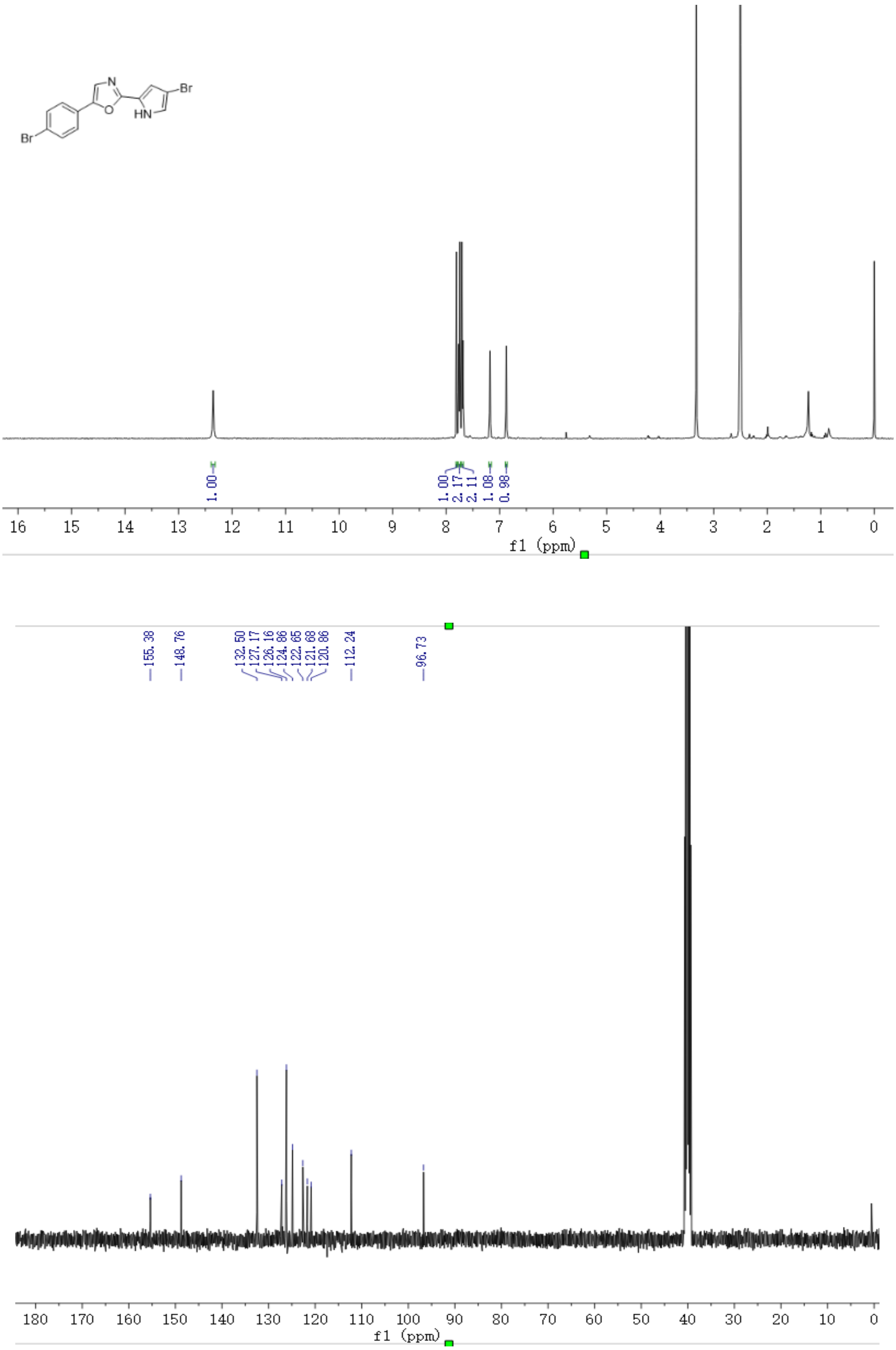

Figure S22. ^{1}H and ^{13}C Spectrum of **23** in DMSO-d_6

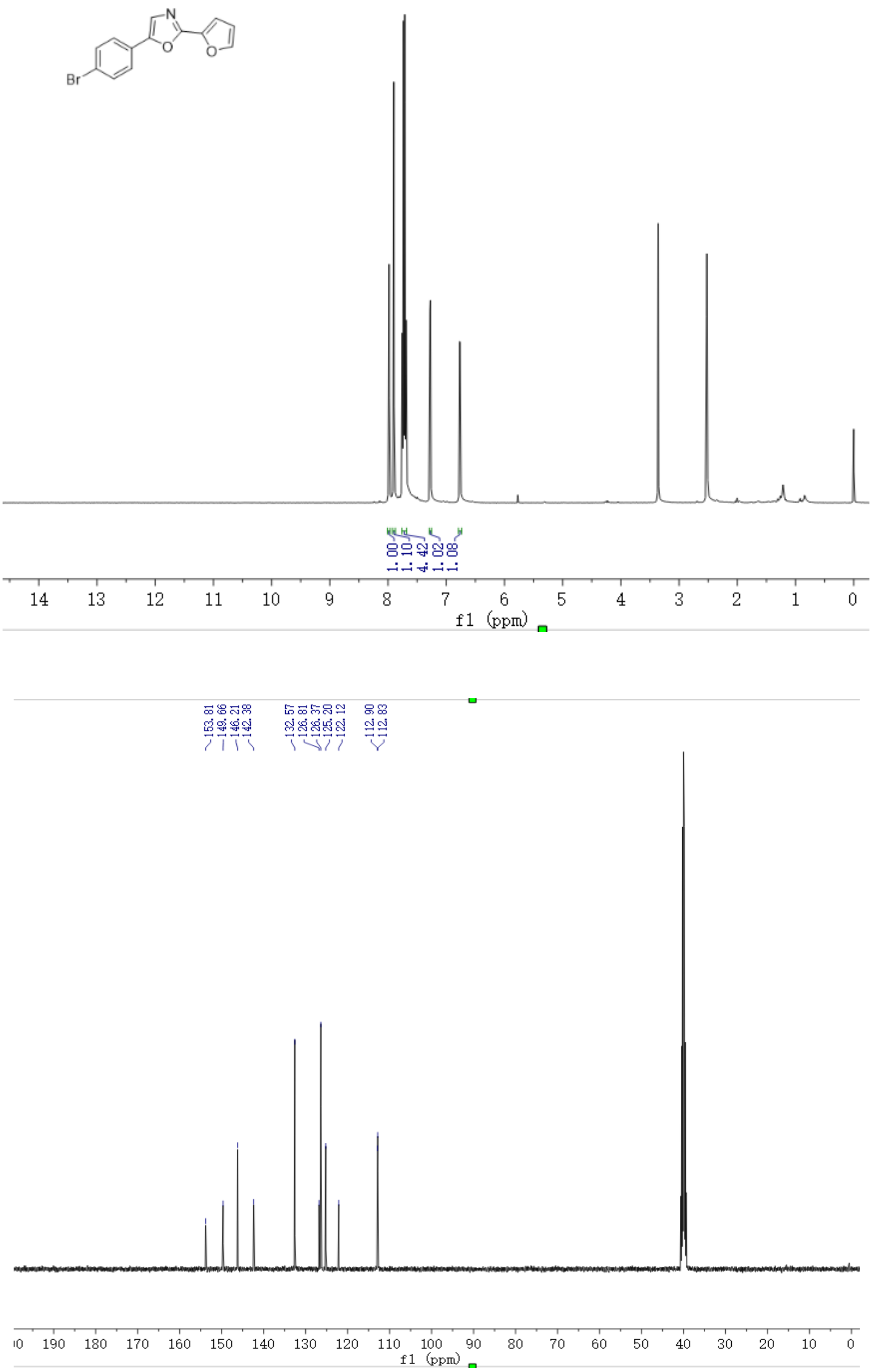

Figure S23. ^{1}H and ^{13}C Spectrum of **24** in DMSO-d_6

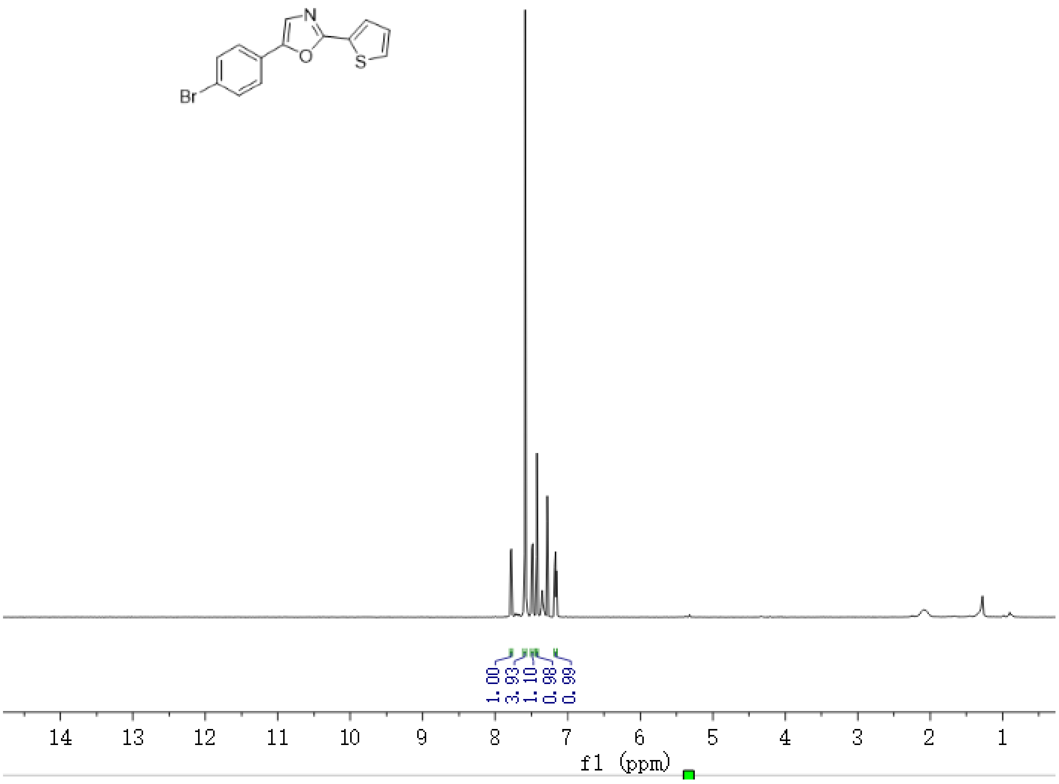

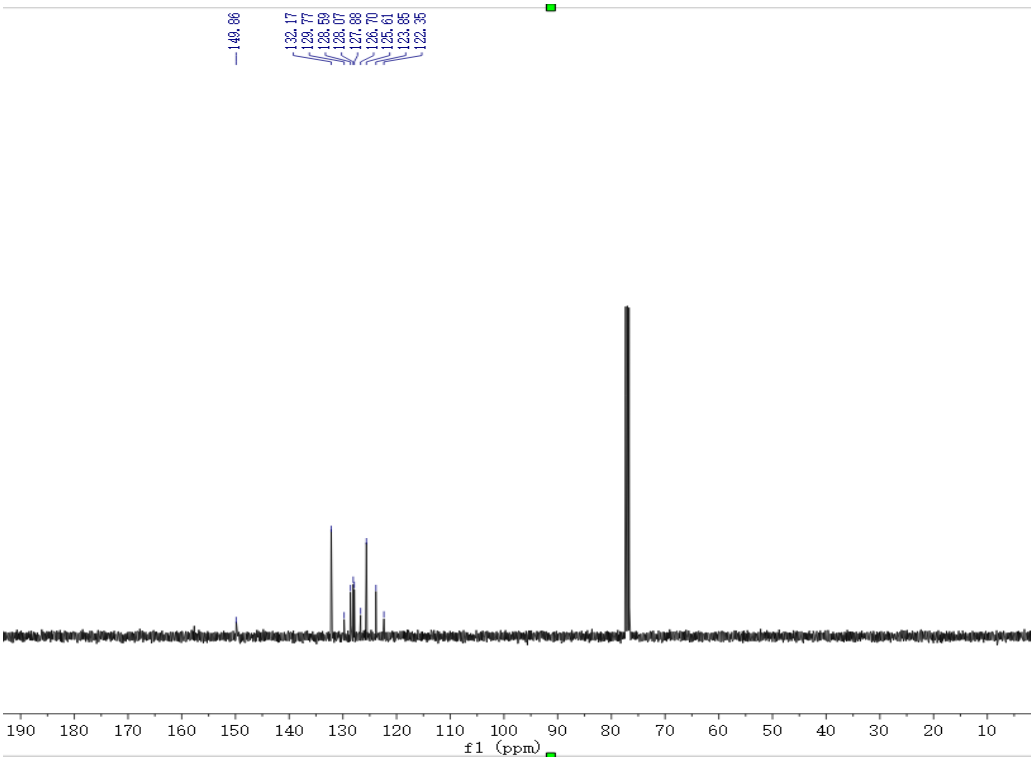

Figure S24. ^{1}H and ^{13}C Spectrum of **25** in DMSO-d_6

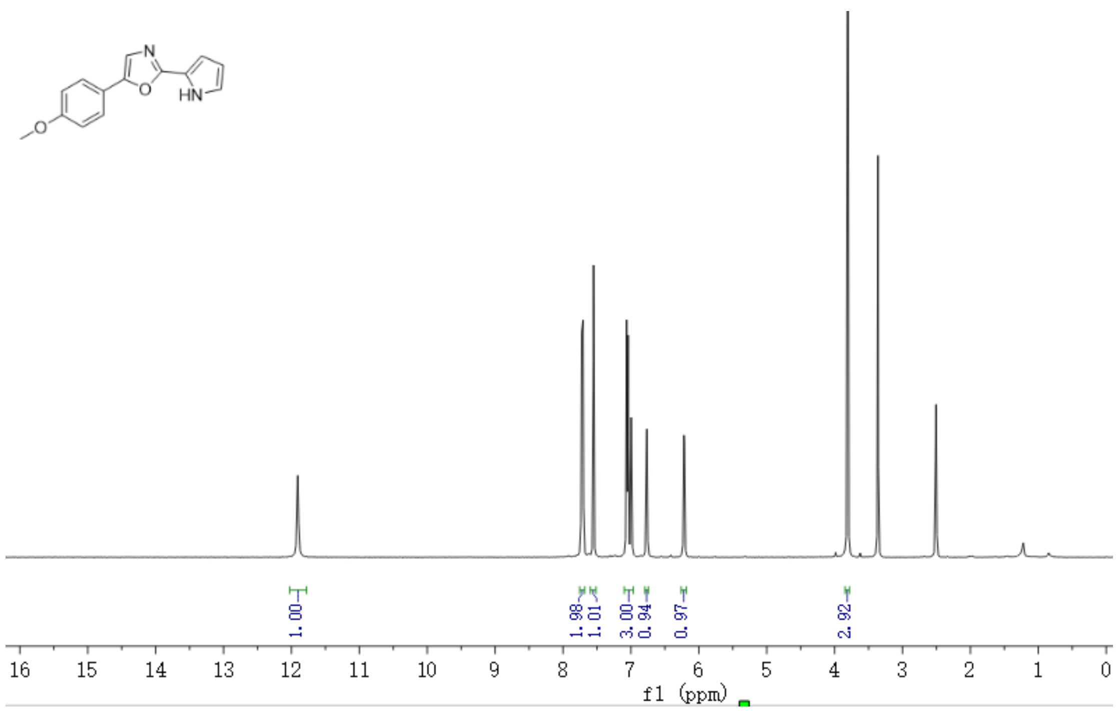

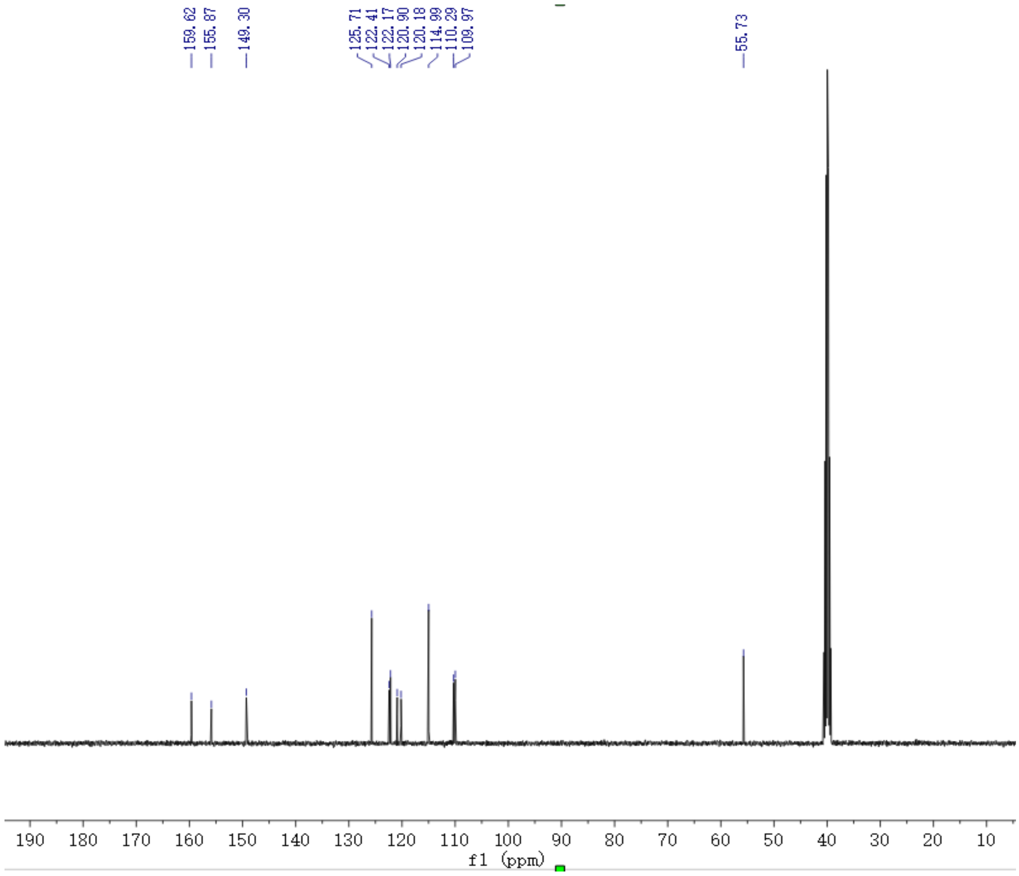

Figure S25. ^{1}H and [13]C Spectrum of **26** in DMSO-d_6

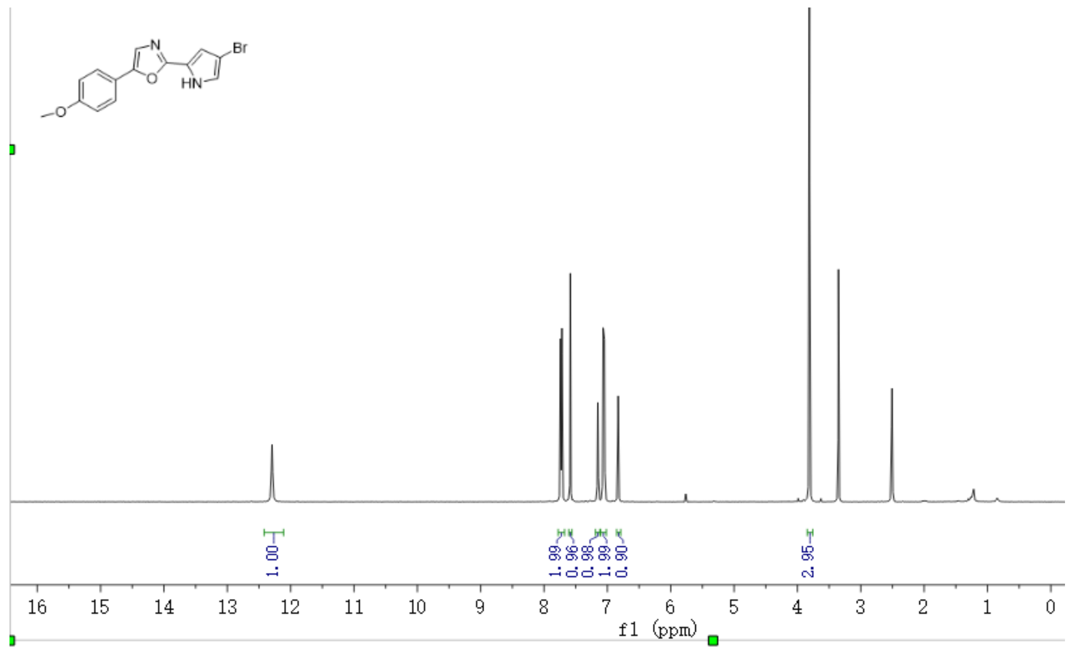

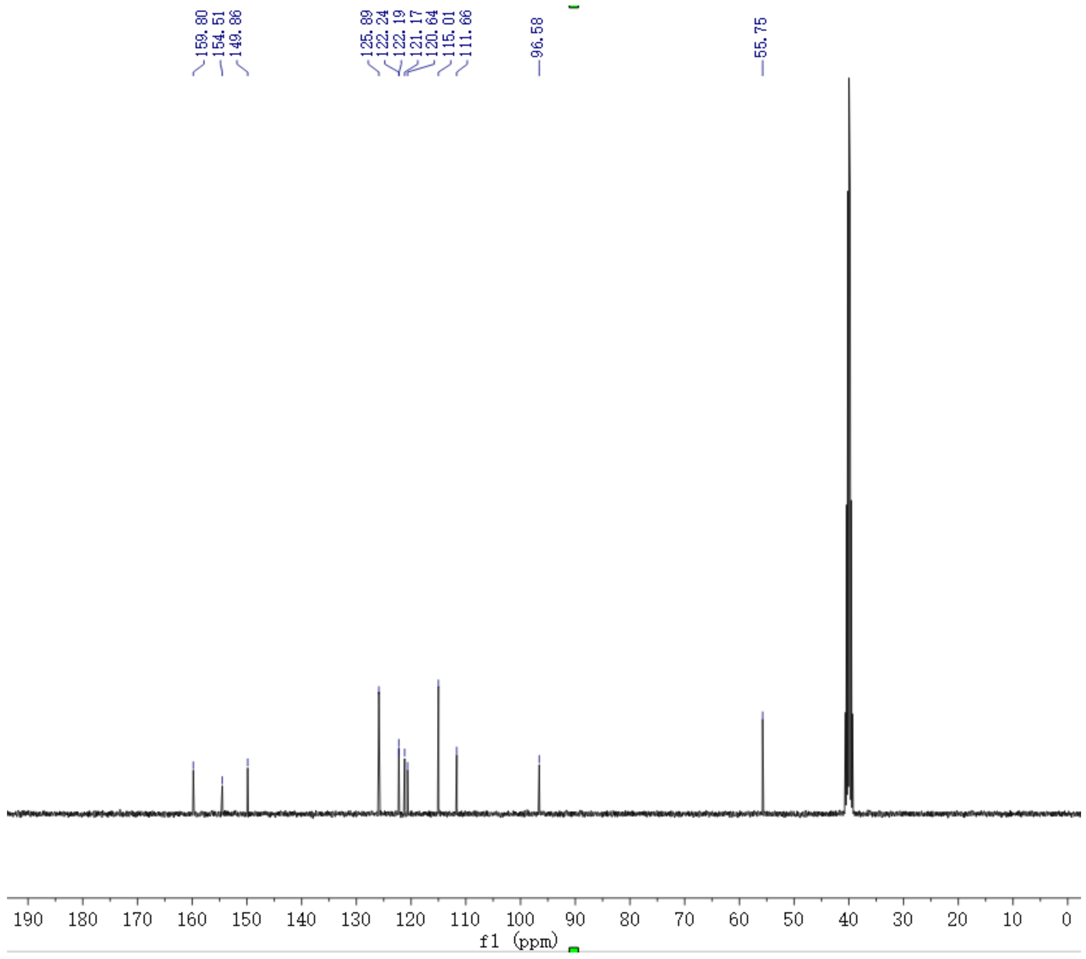

Figure S26. ^{1}H and ^{13}C Spectrum of **27** in DMSO-d_6

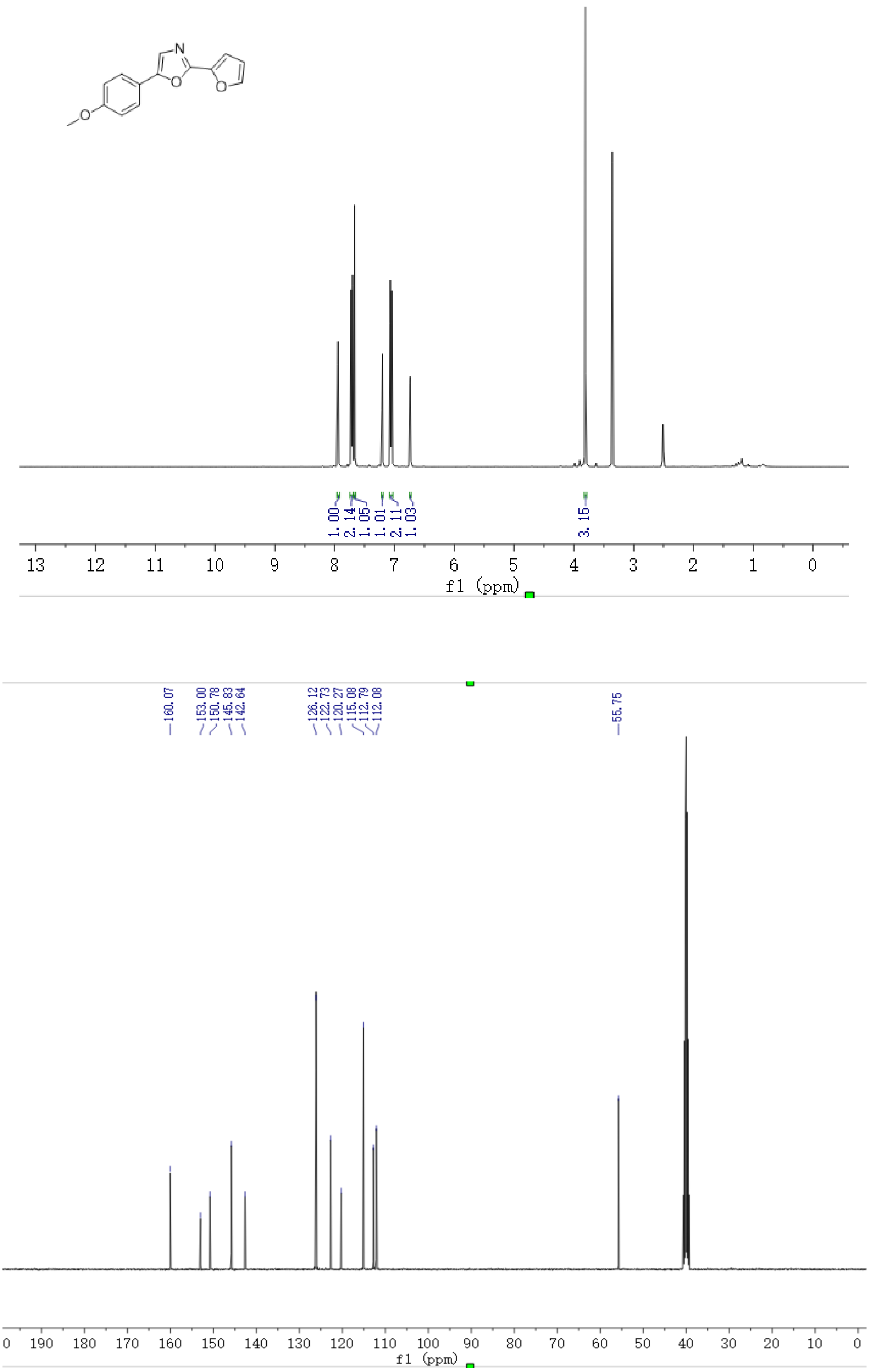

Figure S27. ^{1}H and ^{13}C Spectrum of **28** in DMSO-d_6

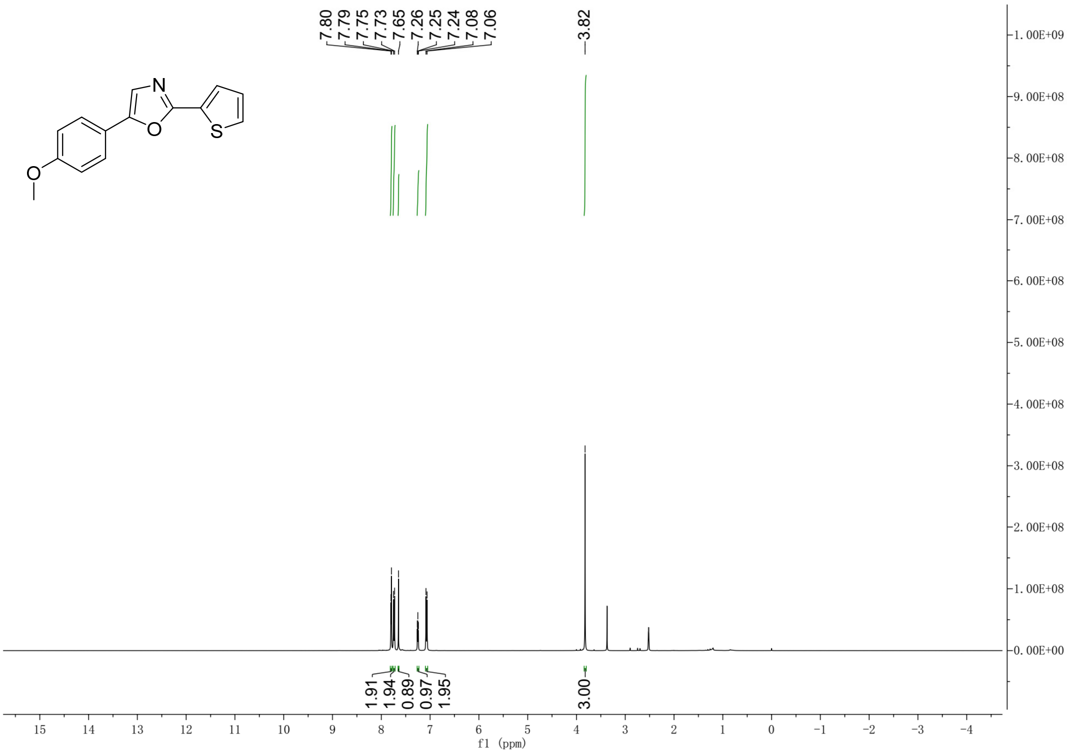

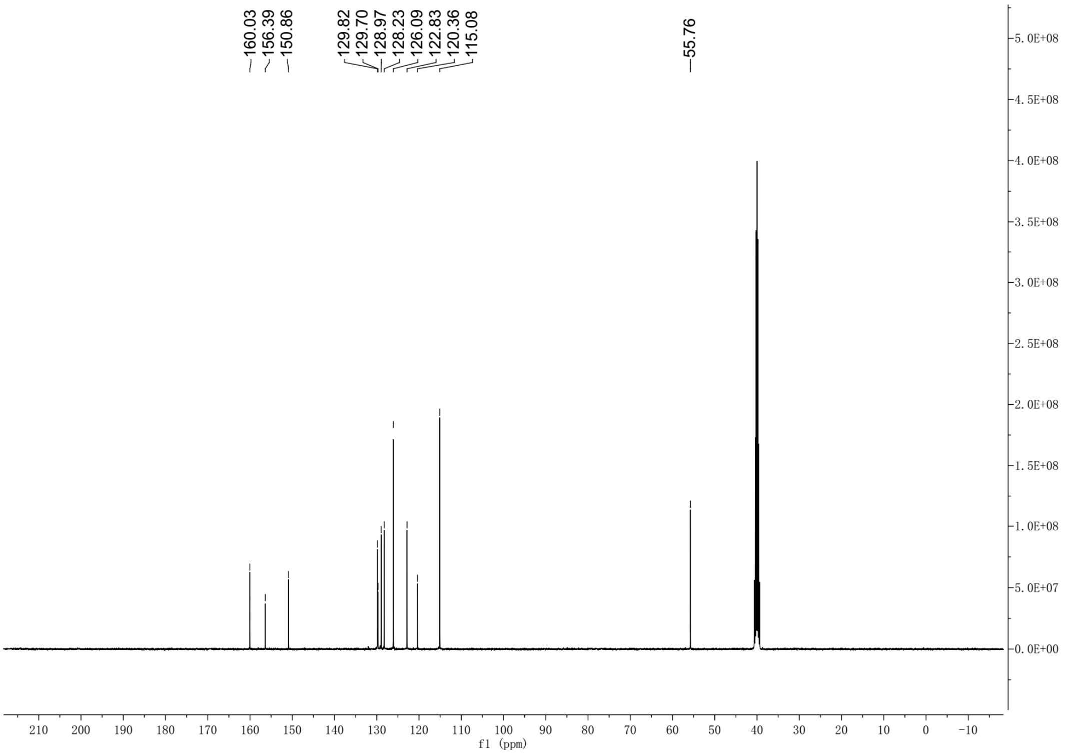

Figure S28. ^{1}H and [13]C Spectrum of **29** in DMSO-d_6
